# Parvalbumin interneuron impairment causes synaptic transmission deficits and seizures in *SCN8A* developmental and epileptic encephalopathy

**DOI:** 10.1172/jci.insight.181005

**Published:** 2024-10-22

**Authors:** Raquel M. Miralles, Alexis R. Boscia, Shrinidhi Kittur, Jessica C. Hanflink, Payal S. Panchal, Matthew S. Yorek, Tyler C. J. Deutsch, Caeley M. Reever, Shreya R. Vundela, Eric R. Wengert, Manoj K. Patel

**Affiliations:** 1 Department of Anesthesiology and; 2 Neuroscience Graduate Program, University of Virginia Health System, Charlottesville, Virginia, USA.; 3 Division of Neurology, Department of Pediatrics, The Children’s Hospital of Philadelphia, Philadelphia, Pennsylvania, USA.

**Keywords:** Neuroscience, Epilepsy, Genetic diseases, Sodium channels

## Abstract

*SCN8A* developmental and epileptic encephalopathy (DEE) is a severe epilepsy syndrome resulting from mutations in the voltage-gated sodium channel Na_v_1.6, encoded by the gene *SCN8A*. Na_v_1.6 is expressed in excitatory and inhibitory neurons, yet previous studies primarily focus on how *SCN8A* mutations affect excitatory neurons, with limited studies on the importance of inhibitory interneurons. Parvalbumin (PV) interneurons are a prominent inhibitory interneuron subtype that expresses Na_v_1.6. To assess PV interneuron function within *SCN8A* DEE, we used 2 mouse models harboring patient-derived *SCN8A* gain-of-function variants, *Scn8a*^D/+^, where the *SCN8A* variant N1768D is expressed globally, and *Scn8a*^W/+^-PV, where the *SCN8A* variant R1872W is selectively expressed in PV interneurons. Expression of the R1872W *SCN8A* variant selectively in PV interneurons led to development of spontaneous seizures and seizure-induced death. Electrophysiology studies showed that *Scn8a*^D/+^ and *Scn8a*^W/+^-PV interneurons were susceptible to depolarization block and exhibited increased persistent sodium current. Evaluation of synaptic connections between PV interneurons and pyramidal cells showed synaptic transmission deficits in *Scn8a*^D/+^ and *Scn8a*^W/+^-PV interneurons. Together, our findings indicate that PV interneuron failure via depolarization block along with inhibitory synaptic impairment likely elicits an overall inhibitory reduction in *SCN8A* DEE, leading to unchecked excitation and ultimately resulting in seizures and seizure-induced death.

## Abstract 2

Miralles et al. reveal novel role of PV interneurons in SCN8A epilepsy, where interneuron-specific mutation expression causes seizures in mice, highlighting their previously underestimated impact.

## Introduction

*SCN8A* developmental and epileptic encephalopathy (DEE) is a genetic epilepsy syndrome characterized by treatment-resistant seizures, developmental delay, cognitive dysfunction, and an increased incidence of sudden unexpected death in epilepsy (SUDEP) (1–4). It is caused by de novo gain-of-function (GOF) mutations in the *SCN8A* gene (5), which encodes the sodium channel Na_v_1.6 (6). Na_v_1.6 is expressed widely in the central nervous system and is prominent at the axon initial segment (AIS) of both excitatory and inhibitory neurons (7–9). Previous studies using mouse models of *SCN8A* DEE show that excitatory neurons are hyperexcitable (10), whereas somatostatin inhibitory interneurons experience increased susceptibility to depolarization block, a mechanism of action potential failure (11). Despite advances in understanding the physiological mechanisms of *SCN8A* DEE, current treatments are often unable to control seizures and reduce the risk of SUDEP, highlighting the need to further understand the underlying network mechanisms of this disorder.

The balance of excitation and inhibition in the brain is critical in seizure generation. Inhibitory interneurons suppress the activity of their target excitatory neurons in an effort to control network dynamics and prevent any excessive excitation that may lead to seizures (12–15). Inhibitory interneurons are incredibly diverse; a recent study has identified 28 subtypes based on morphological, electrophysiological, and transcriptomic data (16). Due to their diversity, classifications of cortical inhibitory interneurons are often changing, but currently there are 5 major identified subclasses: parvalbumin (PV), somatostatin (SST), vasoactive intestinal peptide (VIP), Lamp5, and Sncg interneurons (14–18). The most numerous subtype is PV interneurons, which make up about 40% of inhibitory interneurons and provide feed-forward and feedback inhibition to networks through reliable, high-frequency firing (14, 15). PV interneurons are known to express relatively high levels of Na_v_1.6 compared with other inhibitory interneurons (19) and yet have been previously unstudied in the context of *SCN8A* DEE, significantly limiting our understanding of the seizure network in this disorder. Inhibitory interneuron dysfunction has been heavily implicated in Dravet syndrome, another sodium channelopathy resulting from mutations in the *SCN1A* gene. Previous studies of Dravet syndrome indicate that PV interneurons are hypoexcitable during a critical developmental time window (20, 21). In adult mice, PV interneurons show deficits in synaptic transmission and synchronization that likely contribute to the chronic phenotype of Dravet syndrome (22, 23). Additionally, PV interneurons have also been implicated in temporal lobe epilepsy (TLE). In mouse models of TLE, previous studies show a reduction in PV staining, indicating a potential loss of PV interneurons (24, 25), and others suggest a role for PV interneurons in abnormal synapse formation (26, 27).

In this study, we used 2 mouse models of *SCN8A* DEE harboring the N1768D (*Scn8a*^D/+^) and R1872W (*Scn8a*^W/+^) patient-derived *SCN8A* variants. These models recapitulate key features of the disease through spontaneous seizures and increased risk of seizure-induced death (28–30). *Scn8a*^D/+^ mice express a germline knockin of the N1768D variant (28, 29), whereas *Scn8a*^W/+^ mice harbor a Cre-dependent knockin of the R1872W variant (30). Previous studies have used this conditional expression model to investigate cell type–specific contributions to *SCN8A* DEE: selective expression of the R1872W variant in forebrain excitatory neurons leads to spontaneous seizures and premature death (30), whereas selective expression of this mutation in SST inhibitory interneurons leads to audiogenic seizures without spontaneous seizures or seizure-induced death (11).

Here, we used both the global *Scn8a*^D/+^ model and the conditional *Scn8a*^W/+^ model of *SCN8A* DEE to assess the phenotype of mutant PV interneurons individually and as a component of the *SCN8A* DEE network. We report that selective expression of the R1872W *SCN8A* variant in PV interneurons (*Scn8a*^W/+^-PV) is sufficient to induce spontaneous seizures and premature seizure-induced death, indicating the importance of this inhibitory subtype to *SCN8A* DEE as a whole. Whole-cell patch clamp electrophysiology recordings of PV interneurons demonstrated an increased susceptibility to action potential (AP) failure via depolarization block. Consequently, we also observed a decrease in spontaneous inhibition received by pyramidal cells in *Scn8a* mutant mice. Recordings of voltage-gated sodium currents showed an elevation of the persistent sodium current (I_NaP_) in both models and an elevation of resurgent sodium current (I_NaR_) in the *Scn8a*^W/+^-PV model, potentially contributing to the depolarization block phenotype. A decrease in miniature inhibitory postsynaptic currents (mIPSCs) generated in *Scn8a*^W/+^-PV pyramidal cells (PCs) was also observed, suggesting a possible synaptic deficit between PV interneuron and PCs (PV:PC pairs), and dual recordings of synaptically connected cells revealed an increase in PV:PC synaptic transmission failure as well as a prolonged synaptic latency. In summary, these data reveal a substantial and previously unappreciated impairment of PV interneurons and their synaptic connections to excitatory PCs in *SCN8A* DEE. Selective expression of an *SCN8A* variant in PV interneurons shows that these impairments are sufficient to cause seizures and SUDEP in mice, indicating the importance of this critical interneuron subtype to seizure generation and redefining our understanding of the cortical microcircuit function in this disease.

## Results

### Spontaneous seizures and seizure-induced death in mice with selective expression of mutant Na_v_1.6 in PV interneurons.

**Figure 1:**
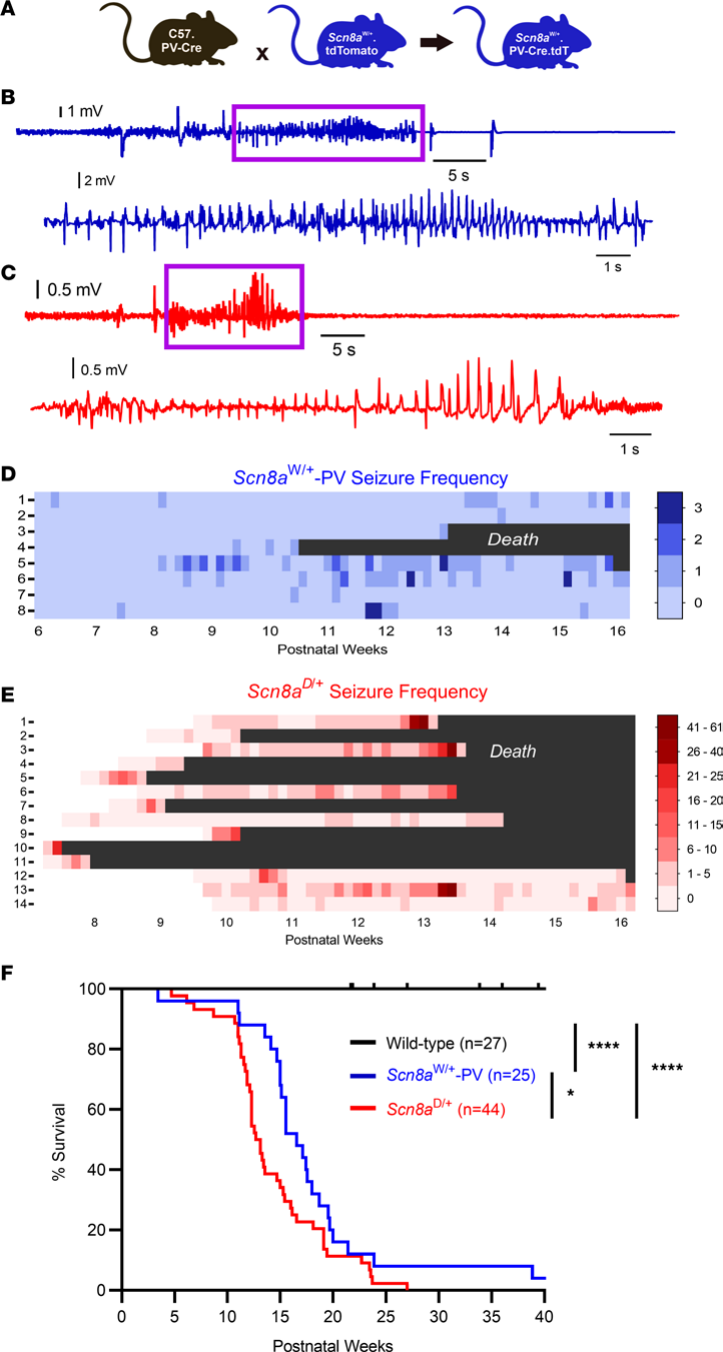
Mice expressing the patient-derived *SCN8A* variant R1872W exclusively in PV interneurons exhibit spontaneous seizures and seizure-induced death. (**A**) Breeding strategy used to produce *Scn8a*^W/+^.tdT.PV-Cre mice (*Scn8a*^W/+^-PV mice, used for both in vivo and whole-cell patch clamp experiments) and age-matched littermate controls on a C57 background. These mice express the R1872W *SCN8A* mutation exclusively in PV interneurons, which are fluorescently labeled with tdTomato. (**B**) Example EEG recording of a spontaneous seizure (shown in blue) from an adult *Scn8a*^W/+^-PV mouse. Spontaneous seizure shown here resulted in seizure-induced death (Supplemental Videos 1 and 2). Purple box highlights spike wave discharges, expanded below. (**C**) Example EEG recording of a spontaneous seizure (red) from an adult *Scn8a*^D/+^ mouse, which expresses the N1768D *SCN8A* variant globally. Purple box highlights spike wave discharges, expanded below. (**D**) Seizure heatmap of (*n* = 8) *Scn8a*^W/+^-PV mice over a period of 10 weeks. (**E**) Seizure heatmap of (*n* = 14) *Scn8a*^D/+^ mice over a period of about 8 weeks. Monitoring began at slightly varying ages, indicated by white in heatmap. (**F**) Survival of *Scn8a*^W/+^-PV mice (*n* = 25) and *Scn8a*^D/+^ mice (*n* = 44) is significantly reduced when compared with WT (*n* = 27; ****, *P* < 0.0001; log-rank Mantel-Cox test). Survival of *Scn8a*^D/+^ mice is decreased compared with *Scn8a*^W/+^-PV mice (*, *P* < 0.05, log-rank Mantel-Cox test).

We first sought to determine if expression of a GOF *SCN8A* variant selectively in PV interneurons would be sufficient for the development of spontaneous seizures. We used the conditional knockin *Scn8a*^W/+^ mouse model and crossed homozygous PV-Cre mice with *Scn8a*^W/+^.tdT mice to generate *Scn8a*^W/+^-PV mice, where the R1872W *SCN8A* variant is expressed exclusively in PV interneurons (Figure 1A). *Scn8a*^W/+^-PV mice were implanted with EEG recording electrodes and monitored for 10 weeks. To better conceptualize the phenotype of *Scn8a*^W/+^-PV mice with reference to another *SCN8A* DEE model, we also implanted EEG recording electrodes in *Scn8a*^D/+^ mice, which express the N1768D *SCN8A* variant globally, and monitored for 6–8 weeks. Spontaneous seizures were observed in all recorded *Scn8a*^W/+^-PV mice (*n* = 8; Figure 1, B and D) and *Scn8a*^D/+^ mice (*n* = 14, Figure 1, C and E). Median seizure onset in *Scn8a*^W/+^-PV mice was approximately 10 weeks of age. In *Scn8a*^W/+^-PV mice, seizures typically consisted of a wild running phase, which was immediately followed by a tonic-clonic phase in approximately 26% of seizures (23/89). Analysis of EEG signals from both *Scn8a*^D/+^ and *Scn8a*^W/+^-PV mice revealed spike wave discharges, a distinct aspect of electrographic seizures (Figure 1, B and C), highlighting similarities between a global mutation model and a model harboring an *SCN8A* variant exclusively in PV interneurons. *Scn8a*^W/+^-PV mice also died prematurely compared with WT littermates, with a median survival of 16.6 weeks (Figure 1F). Electrographic and video recordings verified *Scn8a*^W/+^-PV mice that died during monitoring succumbed to seizure-induced death (*n* = 3; Supplemental Videos 1 and 2). Interestingly, all fatal seizures exhibited a tonic phase before death, consistent with our previous findings in *SCN8A* DEE mice (31). In agreement with previous studies (29), *Scn8a*^D/+^ mice also died prematurely as a result of seizure-induced death (Figure 1F and Supplemental Video 3), which was significantly accelerated compared with *Scn8a*^W/+^-PV mice (*P* = 0.024). Overall, these findings show that a GOF variant exclusively expressed in PV interneurons can lead to seizures and seizure-induced death and support a previously unappreciated role for PV interneurons in seizure induction and seizure-induced death in a mouse model of *SCN8A* DEE.

### Depolarization block in Scn8a mutant PV interneurons.

**Figure 2:**
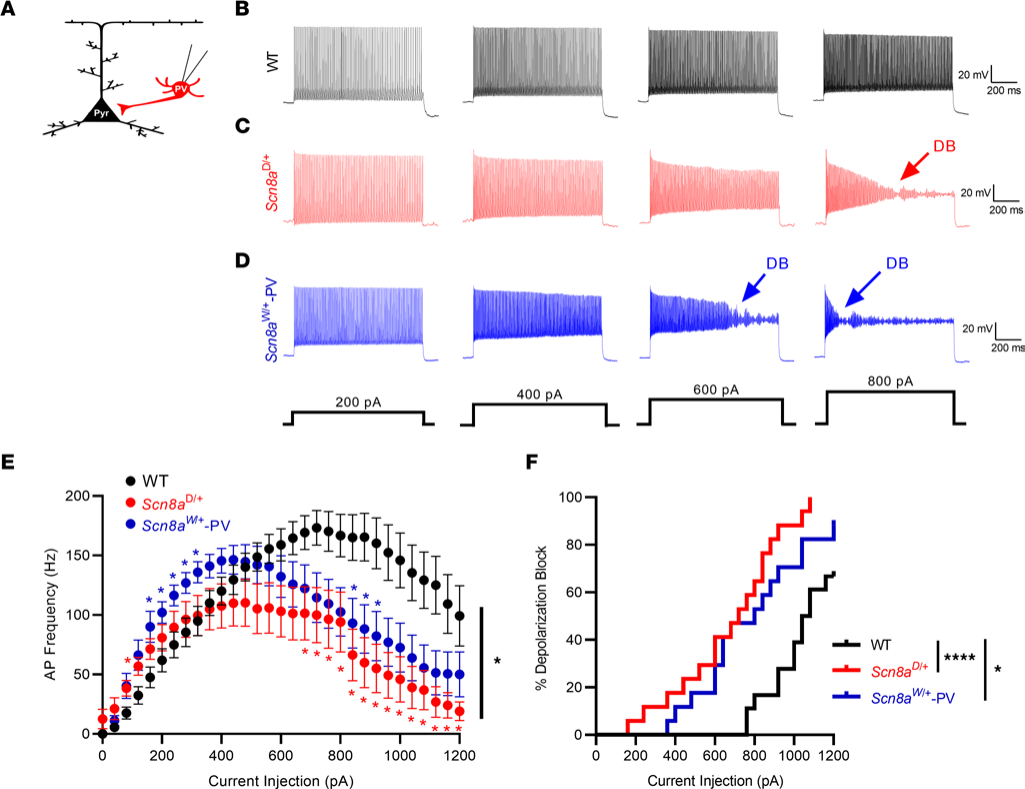
Altered excitability and depolarization block in *Scn8a*^D/+^ and *Scn8a*^W/+^-PV interneurons. (**A**) Whole-cell recordings were collected from WT, *Scn8a*^D/+^, and *Scn8a*^W/+^-PV interneurons in layer IV/V of the somatosensory cortex in adult 5- to 8-week old mice. (**B**–**D**) Example traces of WT (**B**, black), *Scn8a*^D/+^ (**C**, red), and *Scn8a*^W/+^ (**D**, blue) PV interneuron firing at 200, 400, 600, and 800 pA current injections. Depolarization block is noted with arrows (DB). (**E**) *Scn8a*^D/+^ (*n* = 17 cells, 6 mice) and *Scn8a*^W/+^ PV (*n* = 17 cells, 5 mice) interneurons experience a decrease in firing via depolarization block (*, *P* < 0.05, 2-way ANOVA with Tukey’s multiple comparisons test) when compared with WT PV interneurons (*n* = 18 cells, 8 mice). Red or blue stars indicate individual points of significance for either *Scn8a*^D/+^ or *Scn8a*^W/+^-PV, respectively, by multiple comparisons test. (**F**) Cumulative distribution of PV interneuron entry into depolarization block relative to current injection magnitude for WT, *Scn8a*^D/+^, and *Scn8a*^W/+^-PV mice (****, *P* < 0.0001; *, *P* < 0.05, log-rank Mantel-Cox test).

To assess the intrinsic physiological function of *Scn8a* mutant PV interneurons, we performed electrophysiological recordings of fluorescently labeled PV interneurons in layer IV/V of the somatosensory cortex of adult (5 to 8 weeks) *Scn8a*^D/+^, *Scn8a*^W/+^-PV, and age-matched WT littermates (Figure 2A). To verify that fluorescently labeled cells were indeed PV positive, we used immunohistochemistry to stain for PV in WT, *Scn8a*^D/+^, and *Scn8a*^W/+^-PV mice with tdTomato as a Cre-dependent fluorescent marker driven by PV-Cre, where we found that more than 95% of cells were both PV and tdTomato positive (Supplemental Figure 1). WT littermates from both *Scn8a*^D/+^ and *Scn8a*^W/+^-PV genotypes did not exhibit any differences in firing frequencies (*P* = 0.656) and were pooled. Analysis of membrane and AP properties revealed that *Scn8a*^D/+^ PV interneurons had decreased downstroke velocity as well as increased AP width when compared with WT (Table 1). Using a series of depolarizing current injection steps to assess intrinsic excitability, we observed a difference in excitability (*P* = 0.028) between WT, *Scn8a*^D/+^, and *Scn8a*^W/+^ PV interneurons. Initially, PV interneurons expressing either *Scn8a* variant were hyperexcitable compared with WT littermates at lower current injection steps (<100 pA in *Scn8a*^D/+^ mice, *P* = 0.045, and <360 pA in *Scn8a*^W/+^-PV mice, *P* = 0.030). However, at higher current injection steps, both *Scn8a*^D/+^ and *Scn8a*^W/+^ PV interneurons exhibited progressive AP failure as a result of depolarization block (>640 pA in *Scn8a*^D/+^ mice, *P* = 0.042; >840 pA in *Scn8a*^W/+^-PV mice, *P* = 0.041; Figure 2, B–F). Both *Scn8a*^D/+^ and *Scn8a*^W/+^ PV interneurons were more prone to depolarization block than their WT counterparts over the range of current injection magnitudes (*P* < 0.0001 and *P* = 0.016, respectively; Figure 2F). Depolarization block of inhibitory interneurons has been previously implicated in seizure-like activity both in vitro and in vivo and has been proposed as a biophysical mechanism underlying approach of seizure threshold (32–37). Here, the early onset of depolarization block in *Scn8a* mutant PV interneurons indicates a PV hypoexcitability phenotype, similar to the phenotypes observed in PV interneurons in GOF *SCN1A* DEE and in SST interneurons in *SCN8A* DEE (11, 32).

Previous studies have shown that excitatory pyramidal neurons in global knockin *Scn8a*^D/+^ mice are hyperexcitable compared with WT, suggesting that a global change in neuronal activity of both inhibitory and excitatory neurons likely contributes to the seizure phenotype (10). To determine if firing is affected in excitatory neurons from *Scn8a*^W/+^-PV mice, which selectively express an *Scn8a* variant in PV interneurons, we recorded the intrinsic excitability of pyramidal neurons from cortical layers IV/V in adult mice (Supplemental Figure 2). Interestingly, we did not observe any differences in the intrinsic excitability of pyramidal neurons between the WT and *Scn8a*^W/+^-PV genotypes (Supplemental Figure 2). This suggests that alterations in the physiology of PV interneurons may be sufficient in facilitating seizures in *SCN8A* DEE. Analysis of AP parameters revealed an increase in input resistance and a decrease in rheobase (Supplemental Figure 2, E and F, and Supplemental Table 1), suggestive of some compensatory changes in excitatory PCs.

Additionally, the role of development is an important consideration in understanding the pathophysiology of *SCN8A* DEE. In Dravet syndrome, differences in PV interneuron intrinsic excitability are observed only during a critical developmental time window (P18–P21) (21). To determine if the same was true for PV interneurons in *SCN8A* DEE, we measured intrinsic excitability at the critical P18–P21 time window (Supplemental Figure 3). Although no differences in intrinsic excitability were observed (Supplemental Figure 3B), there were significant differences in AP waveform between WT, *Scn8a*^D/+^, and *Scn8a*^W/+^-PV interneurons at P18–P21. APs in P18–P21 *Scn8a* mutant mice were significantly wider, with slower upstroke and downstroke velocities, than in their WT counterparts (Supplemental Figure 3, C–F, and Supplemental Table 2). These findings indicate early alterations in PV interneuron AP parameters before the onset of spontaneous seizures and may suggest a progression of PV interneuron physiology into adulthood.

### GOF Na_v_1.6 mutations affect sodium channel currents in PV interneurons.

**Figure 3:**
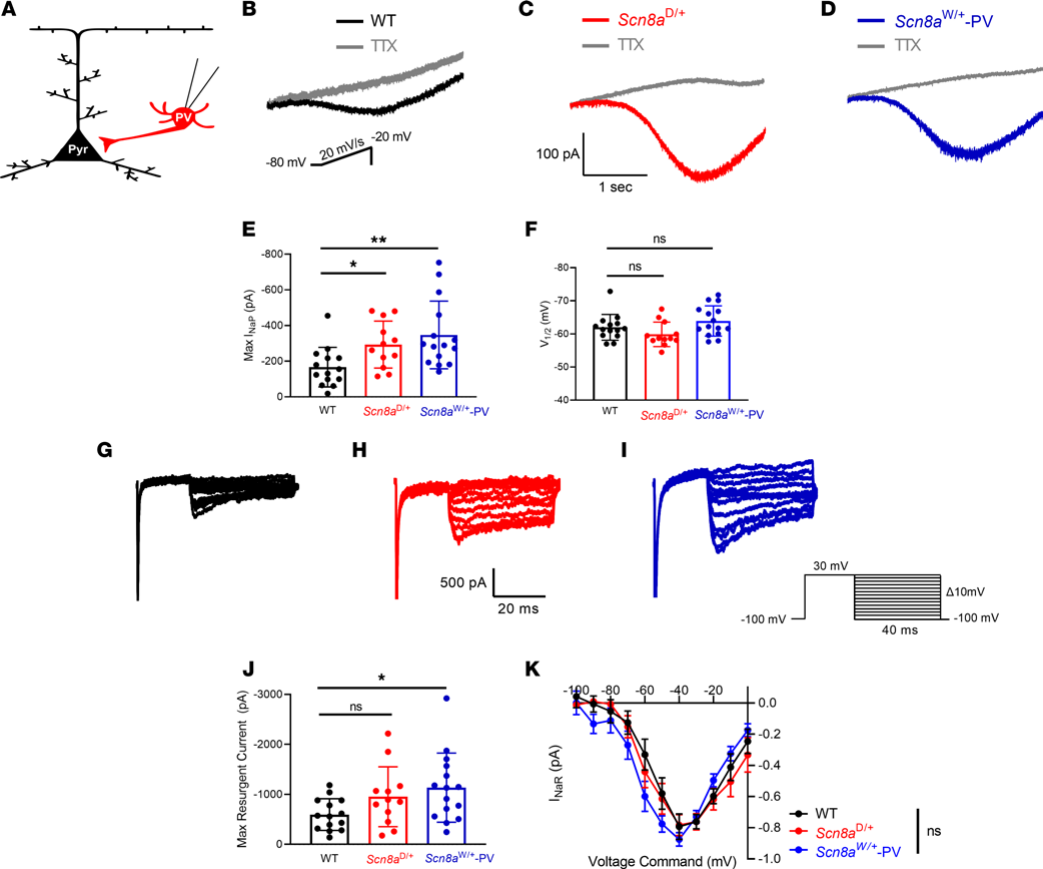
I_NaP_ and I_NaR_ in WT, *Scn8a*^D/+^, and *Scn8a*^W/+^ PV interneurons. (**A**) I_NaP_ and I_NaR_ were recorded via whole-cell patch clamp onto PV interneurons in layer IV/V of the somatosensory cortex in adult, 5- to 8-week-old WT (*n* = 14 cells, 5 mice), *Scn8a*^D/+^ (*n* = 12 cells, 4 mice), and *Scn8a*^W/+^-PV mice (*n* = 15 cells, 5 mice). (**B**–**D**) Example traces of steady-state I_NaP_ evoked by slow voltage ramps from WT (**B**, black), *Scn8a*^D/+^ (**C**, red), and *Scn8a*^W/+^ (**D**, blue) PV interneurons. Traces in gray show slow voltage ramp in the presence of 500 nM tetrodotoxin (TTX). (**E**) Elevated maximum I_NaP_ in *Scn8a*^D/+^ (*, *P* < 0.05) and *Scn8a*^W/+^-PV (**, *P* < 0.01) interneurons compared with WT PV interneurons (Kruskal-Wallis test with Dunn’s multiple comparison test). (**F**) V_1/2_ values were not different between WT, *Scn8a*^D/+^, and *Scn8a*^W/+^-PV mice (*P* > 0.05, 1-way ANOVA with Dunnett’s multiple comparison test). (**G**–**I**) Example traces of TTX-subtracted I_NaR_ for WT (**G**, black), *Scn8a*^D/+^ (**H**, red), and *Scn8a*^W/+^ (**I**, blue). (**J**) Maximum I_NaR_ magnitude was increased between WT and *Scn8a*^W/+^-PV interneurons (*, *P* < 0.05), whereas I_NaR_ magnitude between WT and *Scn8a*^D/+^ PV interneurons was not significantly different (*P* > 0.05, Brown-Forsythe ANOVA with Dunnett’s multiple comparison test). (**K**) Current-voltage relationship for I_NaR_ is not significantly different between WT, *Scn8a*^D/+^, and *Scn8a*^W/+^-PV mice (*P* > 0.05, 2-way ANOVA).

Depolarization block in *Scn8a*^D/+^ and *Scn8a*^W/+^ PV interneurons likely arises from abnormal sodium channel activity as a result of the GOF variant, contributing to changes in membrane depolarization levels and subsequent sodium channel availability for AP initiation. Increases in the I_NaP_ have been identified as a major factor in many epileptic encephalopathy–causing variants, including both the N1768D and R1872W variants in *SCN8A* DEE (5, 10, 30, 38). Further, I_NaP_ is a known determinant of depolarization block threshold (11). In view of this, we recorded I_NaP_ in PV interneurons in the whole-cell patch clamp configuration (Figure 3A). I_NaP_ was increased in both *Scn8a*^D/+^ (–293.1 ± 38.0 pA; *P* = 0.032) and *Scn8a*^W/+^ (–347.1 ± 49.0 pA; *P* = 0.004) PV interneurons when compared with WT (–166.6 ± 29.7 pA; Figure 3, B–E). Half-maximal voltage of activation (V_1/2_) did not differ from WT (–62.0 ± 1.0 mV) in either *Scn8a*^D/+^ (–59.9 ± 1.1 mV; *P* = 0.329) or *Scn8a*^W/+^-PV (–63.9 ± 1.2 mV; *P* = 0.592) mice (Figure 3F). Another component of the sodium current that may affect excitability particularly in fast spiking cells is the I_NaR_ (39, 40). I_NaR_ is a slow inactivating depolarizing current that can contribute to increased AP frequency by providing additional depolarization during the falling phase of an AP (39–41). I_NaR_ has been previously implicated in TLE as well as in sodium channelopathies (42, 43). I_NaR_ was significantly increased in *Scn8a*^W/+^-PV interneurons (–1,136.0 ± 178.5 pA; *P* = 0.037), and while we observed an increasing trend, I_NaR_ was not significantly increased in *Scn8a*^D/+^ PV interneurons (–952.8 ± 172.9 pA; *P* = 0.219), when compared to WT (–595.9 ± 84.8 pA) PV interneurons (Figure 3, G–J). Current-voltage relationship of I_NaR_ was not different between WT, *Scn8a*^D/+^, and *Scn8a*^W/+^-PV mice (*P* = 0.631; Figure 3K). These results demonstrate an increase in 2 components of the overall sodium current in PV interneurons, which possibly contributes to their initial hyperexcitability and increased susceptibility to depolarization block. Increases in both I_NaP_ and I_NaR_ probably provide a sustained level of depolarization, resulting in the accumulation of inactivated sodium channels and increased susceptibility to depolarization block (11, 32, 44).

**Figure 4:**
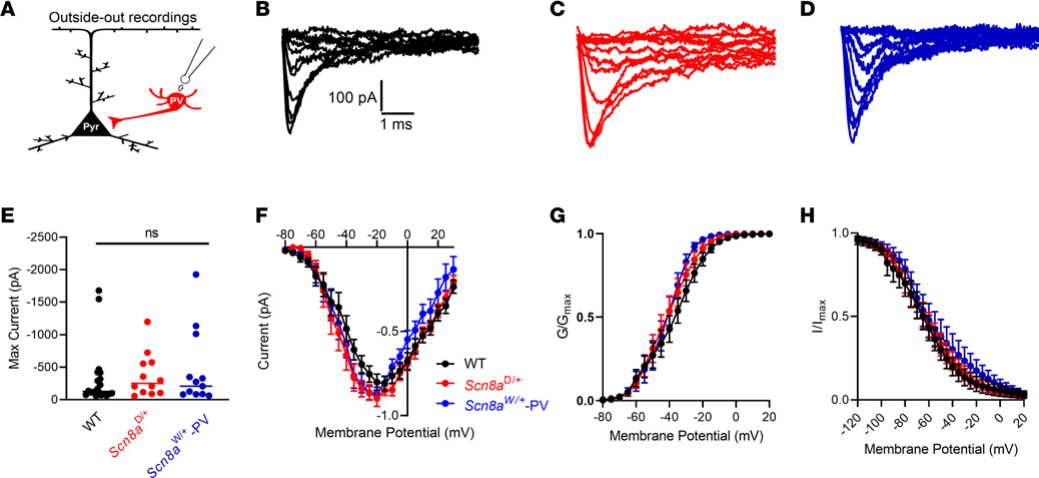
Transient sodium currents in WT, *Scn8a*^D/+^, and *Scn8a*^W/+^ PV interneurons. (**A**) Transient sodium current was assessed in PV interneurons using patch-clamp recordings in the outside-out configuration. (**B**–**D**) Example traces of sodium currents recorded from WT (**B**, black), *Scn8a*^D/+^ (**C**, red), and *Scn8a*^W/+^ (**D**, blue) PV interneurons. (**E**) Maximum transient sodium current was not significantly different between WT (*n* = 20 cells, 8 mice), *Scn8a*^D/+^ (*n* = 12 cells, 4 mice), and *Scn8a*^W/+^ (*n* = 12 cells, 4 mice) PV interneurons (*P* > 0.05, Kruskal-Wallis test with Dunn’s multiple comparison test). (**F**) Current-voltage relationship does not differ between WT, *Scn8a*^D/+^, and *Scn8a*^W/+^-PV interneurons (*P* > 0.05, 2-way ANOVA). (**G**) Voltage-dependent conductance curve does not differ significantly between WT, *Scn8a*^D/+^, and *Scn8a*^W/+^ PV interneurons (*P* > 0.05, 2-way ANOVA). (**H**) Steady-state inactivation does not differ significantly between WT (*n* = 12 cells, 4 mice), *Scn8a*^D/+^ (*n* = 11 cells, 4 mice), and *Scn8a*^W/+^ (*n* = 10 cells, 4 mice) PV interneurons (*P* > 0.05, 2-way ANOVA). Boltzmann curves shown are the average of individual curves generated from fits to data points.

Alterations of both activation and steady-state inactivation parameters of the transient sodium channel current have been previously reported in cells expressing GOF *SCN8A* variants (5, 45–47). To examine PV interneuron sodium channel currents, we performed excised somatic patches in the outside-out configuration from PV interneurons (Figure 4A). Sodium current density, voltage-dependent activation, or steady-state inactivation were not different between WT, *Scn8a*^D/+^, and *Scn8a*^W/+^-PV mice (Figure 4, B–H, and Table 2).

### Decreased inhibitory input onto excitatory neurons in Scn8a mutant mice.

**Figure 5:**
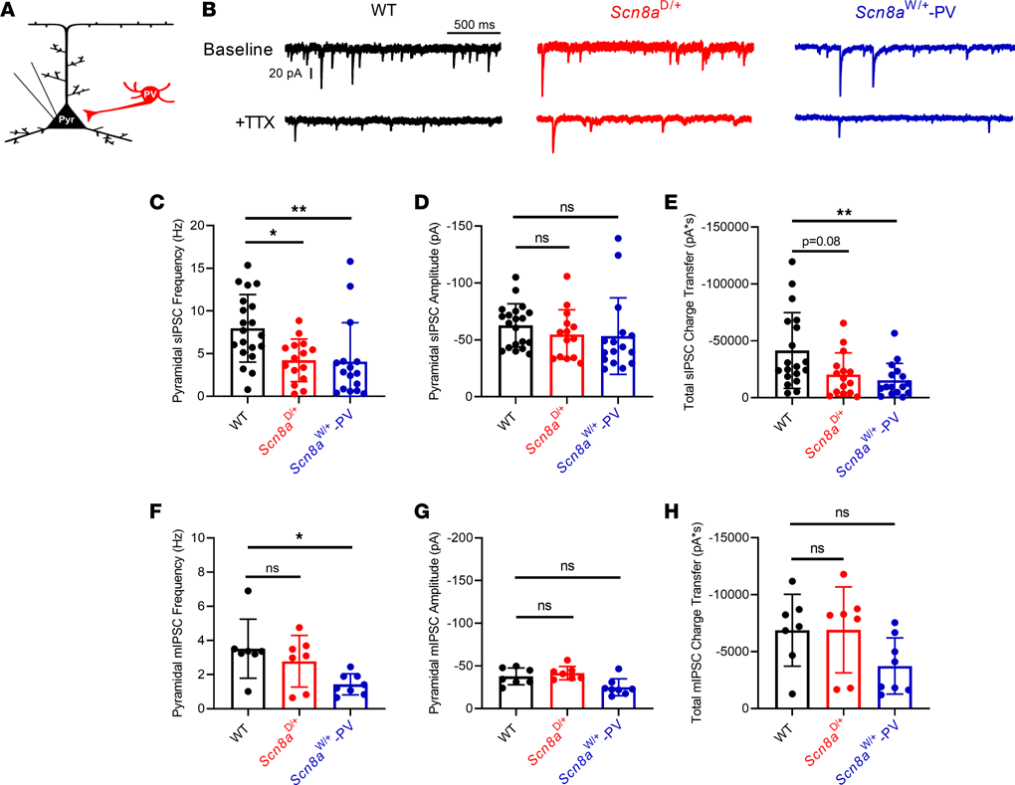
IPSCs generated in PCs from WT, *Scn8a*^D/+^, and *Scn8a*^W/+^-PV mice. (**A**) Whole-cell recordings of IPSCs were collected from cortical layer V PCs in adult, 5- to 8-week-old WT, *Scn8a*^D/+^, and *Scn8a*^W/+^-PV mice. (**B**) Example traces of IPSCs generated in PCs from WT (black), *Scn8a*^D/+^ (red), and *Scn8a*^W/+^-PV (blue) mice. (**C**) Frequency of sIPSCs generated in PCs is decreased in *Scn8a*^D/+^ (*n* = 15 cells, 4 mice, *, *P* < 0.05) and *Scn8a*^W/+^-PV (*n* = 16 cells, 5 mice, **, *P* < 0.01) mice when compared with WT (*n* = 20 cells, 6 mice, Kruskal-Wallis test with Dunn’s multiple-comparison test). (**D**) Amplitude of sIPSCs generated in PCs is not significantly different between groups (*P* > 0.05, Kruskal-Wallis test with Dunn’s multiple-comparison test). (**E**) Total sIPSC charge transfer onto PCs was significantly decreased in *Scn8a*^W/+^-PV mice (**, *P* < 0.01), whereas total sIPSC charge transfer in *Scn8a*^D/+^ mice was not significantly different (*P* > 0.05, Kruskal-Wallis test with Dunn’s multiple-comparison test). (**F**) Frequency of mIPSCs recorded from PCs is decreased in *Scn8a*^W/+^-PV (*n* = 8 cells, 3 mice) mice when compared with WT (*n* = 7 cells, 3 mice, *, *P* < 0.05), whereas frequency of mIPSCs recorded from PCs in *Scn8a*^D/+^ (*n* = 7 cells, 3 mice) mice did not significantly differ from WT (*P* > 0.05, 1-way ANOVA with Dunnett’s multiple-comparison test). (**G**) Amplitude of mIPSCs recorded from PCs is not significantly different between WT, *Scn8a*^D/+^, and *Scn8a*^W/+^-PV mice (*P* > 0.05, Brown-Forsythe ANOVA with Dunnett’s multiple comparison test). (**H**) Total mIPSC charge transfer onto PCs did not significantly differ between WT, *Scn8a*^D/+^, and *Scn8a*^W/+^-PV mice (*P* > 0.05, Kruskal-Wallis test with Dunn’s multiple-comparison test).

Impaired excitability in *Scn8a* mutant PV interneurons may lead to decreased inhibition onto excitatory PCs, as PV interneurons are known to directly inhibit PCs at the soma or AIS (14, 15). To examine how alterations in PV interneuron excitability affect the cortical network, we recorded sIPSCs and mIPSCs from PCs (Figure 5A) as a functional indicator of PV interneuron activity and connectivity. We found that PCs generated significantly fewer sIPSCs in both *Scn8a*^D/+^ (4.22 ± 0.64 Hz; *P* = 0.035) and *Scn8a*^W/+^-PV (4.07 ± 1.14 Hz; *P* = 0.003) mice than their WT counterparts (7.97 ± 0.88 Hz; Figure 5, B and C), suggesting a decrease in inhibitory input onto PCs. sIPSC frequencies between *Scn8a*^D/+^ and *Scn8a*^W/+^-PV PCs were not different (*P* > 0.99), which may imply that PV interneurons are largely responsible for the decrease in somatic inhibitory input in the global *Scn8a*^D/+^ model. sIPSC amplitude was not different between WT (–62.7 ± 4.3 pA), *Scn8a*^D/+^ (–54.7 ± 5.8 pA), and *Scn8a*^W/+^-PV mice (–53.3 ± 8.4 pA; *P* = 0.09, Figure 5D). Additionally, we calculated the total charge transfer from sIPSCs in WT, *Scn8a*^D/+^, and *Scn8a*^W/+^-PV PCs and found that the total spontaneous charge transfer onto PCs was significantly decreased in *Scn8a*^W/+^-PV mice (–15,346 ± 3,706 pA × s; *P* = 0.008) compared with WT (–41,468 ± 7,641 pA × s, Figure 5E). Although it was not statistically significant, we also observed a decreasing trend in spontaneous inhibitory charge transfer in *Scn8a*^D/+^ mice (–20,424 ± 4,895 pA × s; *P* = 0.08; Figure 5E). sIPSC recordings include both AP-induced synaptic transients as well as mIPSCs, which occur because of spontaneous vesicle fusion in the absence of an AP (48, 49). To isolate AP-independent events, we performed recordings in the presence of TTX (500 nM). Relative to WT controls (3.52 ± 0.65 Hz), we found no significant difference in PC mIPSC frequency in *Scn8a*^D/+^ mice (2.78 ± 0.57 Hz; *P* = 0.821), but we did observe a significant reduction of mIPSC frequency in *Scn8a*^W/+^-PV mice (1.43 ± 0.22 Hz; *P* = 0.027; Figure 5F), which could underlie impaired synaptic transmission in *Scn8a*^W/+^-PV mice. mIPSC amplitude did not differ between WT (–37.7 ± 3.7 pA), *Scn8a*^D/+^ (–41.6 ± 3.0 pA; *P* = 0.667), and *Scn8a*^W/+^-PV mice (–25.1 ± 3.5 pA; *P* = 0.055, Figure 5G), though we did observe a decreasing trend in the mIPSC amplitude for *Scn8a*^W/+^-PV mice. Interestingly, we did not observe any significant differences in mIPSC total charge transfer between WT (–6,874 ± 1,194 pA × s), *Scn8a*^D/+^ (–6,907 ± 1,426 pA × s; *P* = 0.984), and *Scn8a*^W/+^-PV mice (–3,734 ± 872.6 pA × s; *P* = 0.133, Figure 5H).

### PV interneuron synaptic transmission is impaired in Scn8a mutant mice.

**Figure 6:**
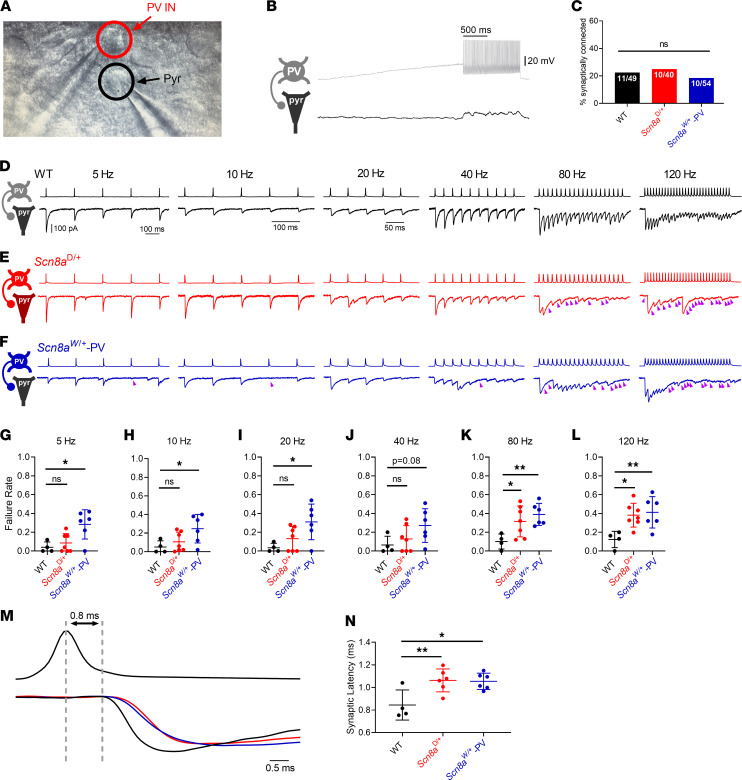
Increased synaptic transmission failure and synaptic latency in *Scn8a* mutant mice. (**A**) Image of dual whole-cell recording of a synaptically connected PV interneuron and pyramidal cell pair. (**B**) Example traces from a PV interneuron (gray) and synaptically coupled pyramidal cell (PC; black). (**C**) Proportion of successfully patched PV:PC pairs that were synaptically connected did not differ between WT (49 pairs from 13 mice), *Scn8a*^D/+^ (40 pairs from 8 mice), and *Scn8a*^W/+^-PV (54 pairs from 9 mice) in adult mice. (**D**–**F**) Example of presynaptic firing and evoked uIPSCs in WT (**D**; black), *Scn8a*^D/+^ (**E**; red), and *Scn8a*^W/+^-PV (**F**; blue) connected pairs at 5 Hz, 10 Hz, 20 Hz, 40 Hz, 80 Hz, and 120 Hz. Purple arrows denote uIPSC failures in the postsynaptic neuron. (**G**–**L**) Summary data for failure rates of evoked uIPSCs at various frequencies. In *Scn8a*^D/+^ connected pairs (*n* = 7, 5 mice), uIPSC failure rate is not significantly different from WT (*n* = 4, 3 mice) at 5, 10, 20, or 40 Hz (*P* > 0.05, **G**–**J**) but is significantly higher at PV interneuron firing frequencies of 80 and 120 Hz (*, *P* < 0.05, **K** and **L**). uIPSC failure rate in *Scn8a*^W/+^-PV pairs (*n* = 6, 5 mice) is significantly higher than WT at 5, 10, 20, 80, and 120 Hz (*, *P* < 0.05, **G**–**I**, **K**, and **L**) but did not significantly differ at 40 Hz (*P* < 0.05, **J**, 1-way ANOVA with Dunnett’s multiple-comparison test). (**M** and **N**) Example traces illustrating synaptic latency in WT, *Scn8a*^D/+^, and *Scn8a*^W/+^-PV, measured from the peak of the presynaptic AP to the onset of the evoked uIPSC (**M**). Gray dotted lines indicate this latency in WT. Latency is increased in *Scn8a*^D/+^ and *Scn8a*^W/+^-PV mice (**N**, 1-way ANOVA with Dunnett’s multiple-comparison test, *, *P* < 0.05, **, *P* < 0.01).

Impairment of synaptic transmission has been suggested as a disease mechanism in multiple epilepsy syndromes, notably Dravet syndrome (23, 50, 51), and proper synaptic signaling is tightly linked to sodium channel function (52). To assess how Na_v_1.6 function influences PV interneuron-mediated inhibitory synaptic transmission, we performed dual whole-cell patch clamp recordings of PV interneurons and nearby PCs to find synaptically connected pairs of cells (Figure 6A). Synaptically connected pairs were identified using a current ramp in the presynaptic PV interneuron to elicit inhibitory postsynaptic potentials (IPSPs) in the postsynaptic PC corresponding to each AP in the PV interneuron (Figure 6B). The number of synaptically connected PV:PC pairs relative to the total number of pairs was not significantly different between WT, *Scn8a*^D/+^, and *Scn8a*^W/+^-PV mice (*P* = 0.634, Figure 6C). In PV:PC connected pairs, we measured the properties of unitary inhibitory postsynaptic currents (uIPSCs) in PCs evoked by stimulation of PV interneurons. To accurately detect uIPSCs, a high-chloride internal solution was used to allow recording of uIPSCs as large inward currents and IPSPs as large membrane depolarizations, overall minimizing the possibility of inaccurately reporting a synaptic failure.

Previous studies indicate that the PV:PC synapse is extremely reliable since PV interneurons have multiple synaptic boutons and a high release probability, indicative of a highly stable synapse (53). PV interneurons are also known to fire reliably at high frequencies (15). We found that stimulation of PV interneurons at a 1 Hz frequency reliably initiated single APs in WT mice. Although we detected some failures in *Scn8a*^D/+^ and *Scn8a*^W/+^-PV mice, there was no significant difference in synaptic failure at a frequency of 1 Hz (*P* = 0.160; Table 3) between the groups, suggesting no deficit in synaptic transmission at low stimulation frequencies. The amplitudes of the uIPSCs also did not differ between genotypes (Table 3, *P* = 0.427). Additionally, to identify any deficits in short-term synaptic plasticity, we used the first 2 IPSCs (IPSC1 and IPSC2) elicited by a presynaptic AP to quantify the paired-pulse ratio (PPR). The PV:PC synapse is known to experience short-term plasticity through synaptic depression (54, 55). We observed synaptic depression in WT, *Scn8a*^D/+^, and *Scn8a*^W/+^-PV connected pairs, with no significant difference in PPR between WT and *Scn8a* mutant pairs (*P* = 0.340 and *P* = 0.189 respectively; Table 3).

To analyze activity-dependent synaptic failure, we then used stimulation trains to elicit multiple APs at increasing frequencies (5, 10, 20, 40, 80, and 120 Hz; Figure 6, D–F, and Table 3). At each frequency, we measured the failure rate of the first and last uIPSC as well as the overall failure rate. Failure rate of the first uIPSC remained low and consistent between WT, *Scn8a*^D/+^, and *Scn8a*^W/+^-PV mice. At lower frequencies (≤40 Hz), there were no differences in overall failure rate or last uIPSC failure rate between WT and *Scn8a*^D/+^mice; however, failure rates were significantly increased in *Scn8a*^W/+^-PV mice at 5, 10, and 20 Hz (Figure 6, G–I), with an increasing trend at a 40 Hz stimulation frequency (Figure 6J). At 80 Hz, the overall failure rate in a 20-pulse train was increased in both *Scn8a*^D/+^ (0.316 ± 0.062; *P* = 0.039) and *Scn8a*^W/+^-PV (0.390 ± 0.048; *P* = 0.009) mice compared with WT (0.101 ± 0.040; Figure 6K), with failures occurring approximately 3 times as frequently in *Scn8a*^W/+^ mice when compared with WT. Similarly, at 120 Hz stimulation frequency with a 30-pulse train, failure rates observed in *Scn8a*^D/+^ and *Scn8a*^W/+^-PV pairs were greater (0.382 ± 0.048 and 0.412 ± 0.068, respectively; *P* = 0.016 and *P* = 0.009) than those observed in their WT counterparts (0.123 ± 0.087; Figure 6L). The progression of total activity-dependent synaptic failure through increasing presynaptic stimulation frequencies is shown in Figure 6, G–L. Additionally, synaptic failure of the last uIPSC in a stimulation train occurred in more than 40% of trials on average with a stimulation frequency of 80 or 120 Hz. We observed that this increase in synaptic failure was significant for the last uIPSC in an 80 Hz train in *Scn8a*^D/+^ (*P* = 0.023) and *Scn8a*^W/+^-PV (*P* = 0.025; Table 3), as well as in a 120 Hz train (*P* = 0.043 and *P* = 0.030, respectively), supporting a greater degree of activity-dependent failure. Analysis of synaptic latency times, measured from the peak of the presynaptic AP to the onset of the postsynaptic uIPSC, revealed an increase in synaptic latency in *Scn8a*^D/+^ (*P* = 0.009) and *Scn8a*^W/+^-PV (*P* = 0.012) mice when compared with WT mice (Figure 6, M and N, and Table 3). Prolonged synaptic latency would suggest an impairment in conduction velocity or GABA release probability, potentially with a longer time lag to vesicle release (56–59). Efficient synaptic transmission and vesicle release are critical for overall network inhibition (60).

## Discussion

PV interneurons prominently express Na_v_1.6 (8, 9) and are known to play a major role in various epilepsies (20, 23–27, 61). However, their role in the pathophysiology of *SCN8A* DEE is unknown. Here, we show that (a) expression of the patient-derived R1872W *SCN8A* GOF variant selectively in PV interneurons conveys susceptibility to spontaneous seizures and premature seizure-induced death; (b) GOF *SCN8A* mutations in PV interneurons lead to initial hyperexcitability and subsequent AP failure via increased susceptibility to depolarization block; (c) PV interneurons in both GOF *SCN8A* mouse models exhibit epileptiform increases in I_NaP_ that would facilitate increased susceptibility to depolarization block; (d) inhibitory input onto excitatory PCs is significantly reduced in *Scn8a* mutant mice; and (e) there is a progressive, activity-dependent increase in synaptic transmission failure from PV inhibitory interneurons onto excitatory neurons. Our findings highlight a role for PV interneurons in the pathophysiology of seizures and seizure-induced death in mouse models of *SCN8A* DEE.

### Expression of R1872W SCN8A mutation in PV interneurons is sufficient to cause seizures and premature death.

PV interneurons are known to be the main drivers for seizure activity in Dravet syndrome, a disorder characterized by deficits in inhibitory neurons, primarily due to haploinsufficiency of Na_v_1.1 (20–23). Selective deletion of Na_v_1.1 in PV interneurons leads to reduced PV interneuron excitability, decreased spontaneous inhibition of excitatory neurons, and increased susceptibility to seizures (61). Similar to impairments observed in mouse models of Dravet syndrome and in *Scn8a*^D/+^ mice, which express the N1768D *SCN8A* variant globally, we show here that selective expression of the GOF R1872W variant in PV interneurons is sufficient to induce spontaneous seizures and leads to seizure-induced death (SUDEP) in mice. Additionally, *Scn8a*^W/+^-PV mice exhibited a reduced seizure frequency and increased survival compared with *Scn8a*^D/+^ mice, and global expression of the R1872W variant or exclusive expression in excitatory neurons leads to a more severe SUDEP phenotype than *Scn8a*^W/+^-PV mice (30). This may indicate that although PV interneurons are an important contributor to the *SCN8A* DEE phenotype, dysfunction of excitatory neurons remains a critical aspect of the disease physiology, as previously published (10, 30, 62). Overall, these findings not only support an important role for PV interneurons in the seizure phenotype of *SCN8A* DEE but also provide support for a major role for Na_v_1.6 channels in controlling PV interneuron excitability in addition to Na_v_1.1 channels.

### GOF SCN8A mutations result in premature PV interneuron depolarization block.

Proper function of *Scn8a* is critical in repetitive firing (41), and as such, we reasoned that mutations affecting the function of *Scn8a* would affect the high-frequency, repetitive firing characteristic of PV interneurons. However, although at lower current injection magnitudes PV interneurons from both mutant mouse models were hyperexcitable, at higher magnitudes we observed PV interneuron AP failure through depolarization block, resulting in overall PV interneuron hypoexcitability. Increased susceptibility to depolarization block due to a GOF sodium channel mutation has been shown previously in both *SCN8A* DEE and *SCN1A* DEE (11, 32). Additionally, depolarization block in PV interneurons leads to hyperactivity and subsequent epileptic discharges in excitatory cells, and rescue of depolarization block via optogenetic stimulation leads to a reduction in epileptiform activity (34–36). Further, in vivo recording of PV interneurons shows evidence for PV depolarization block during seizure activity (37).

Interestingly, the susceptibility to depolarization block and subsequent hypoexcitability in inhibitory interneurons reported here indicates that a mechanism for seizures in *SCN8A* DEE, a disorder characterized primarily by GOF sodium channel mutations, shares many similarities to that of Dravet syndrome, a disorder primarily characterized by sodium channel haploinsufficiency in inhibitory neurons. A study using a model of Dravet syndrome showed a similar pattern of initial hyperexcitability in PV interneurons followed by depolarization block (63). However, impairment of PV interneuron excitability in Dravet syndrome is specific to the P18–P21 developmental time window (21), whereas in *SCN8A* DEE, PV interneuron activity is more markedly impaired in adulthood. The initial hyperexcitability of inhibitory interneurons seen in both *SCN8A* DEE and Dravet syndrome may play some role in the shared comorbidities between these severe developmental disorders.

### Impaired synaptic transmission between mutant PV interneurons and PCs.

We believe our study is the first to examine alterations in synaptic transmission between PV interneurons and excitatory neurons in *SCN8A* DEE, and we show a distinct impairment of inhibitory synaptic transmission onto excitatory PCs in 2 patient-derived mutation models. Synaptic transmission was impaired in both *Scn8a* mutant mouse models: *Scn8a*^W/+^-PV connected pairs failed substantially more than WT at most frequencies, whereas *Scn8a*^D/+^ pairs failed in an activity-dependent manner. Considering the fast-spiking nature of PV interneurons and the degree of inhibitory input they provide on neuronal excitatory networks, activity-dependent failure alone could have a substantial impact on overall seizure susceptibility. A likely mechanism for this failure could be impaired AP propagation, as proper signaling from PV interneurons requires a specific density and function of sodium channels (52). This is further supported by the observed increase in synaptic latency, indicating that propagation may be slowed in *Scn8a* mutant mice. Similarly, synaptic transmission between PV interneurons and PCs is also impaired in Dravet syndrome, though unlike our findings in *SCN8A* DEE, intrinsic excitability deficits are restored in adult PV interneurons (21, 23). A limitation of the study is the number of synaptically connected pairs recorded. It is possible that synaptic transmission is impaired in a non-activity-dependent manner in both *Scn8a*^D/+^ and *Scn8a*^W/+^-PV mice, as a slight increasing trend in the failure rates of *Scn8a*^D/+^ uIPSCs at low frequencies was observed.

Both depolarization block and synaptic transmission failure occurred at high PV interneuron firing frequencies, and as such, it is important to consider in vivo firing frequencies of PV interneurons. PV interneurons are a heterogeneous group made up of primarily basket cells and chandelier cells, which are named for their unique morphologies. These subtypes have slightly different firing patterns and synaptic targets (14, 15). Since our recordings are focused within cortical layers IV/V, it is likely that we recorded primarily from PV-positive basket cells rather than chandelier cells. In vivo, PV interneurons, particularly basket cells, are phase-locked to gamma oscillations, which typically occur between 40 and 100 Hz (64). Events such as sharp wave ripples (SWRs) can lead to PV firing frequencies of more than 120 Hz in vivo (65). This demonstrates the relevance of both increased susceptibility of PV interneurons to depolarization block and of PV:PC synaptic transmission failure at high frequencies with expression of mutant *Scn8a*. These gamma oscillations and SWRs are most often associated with the hippocampus; however, there is evidence for oscillations in the cortex (66, 67). Recently, SWRs have been associated with epileptic discharges in Dravet syndrome: an increase in SWR amplitude may lead to inhibitory depolarization block and a shift into seizure-like activity (68). Considering the frequencies at which we observe PV interneuron failure in both *Scn8a* mutant mouse models, increased susceptibility to depolarization block and failure of inhibitory synaptic transmission could underlie an additional mechanism of seizure generation in *SCN8A* DEE.

### Elevated sodium currents in Scn8a mutant PV interneurons.

We observed an increase in I_NaP_ in both *Scn8a*^D/+^ and *Scn8a*^W/+^-PV interneurons with an increase in I_NaR_ in *Scn8a*^W/+^-PV interneurons. However, we observed no difference in the transient sodium current in *Scn8a*^D/+^ and *Scn8a*^W/+^-PV interneurons, though it is possible that excised somatic patches may not have recapitulated the high levels of Na_v_1.6 in the axon. Previous studies suggest that *Scn8a* may have a much larger role in I_NaR_ than transient current (41). Increases in I_NaP_ have been implicated in various epilepsies (38, 42, 44, 69), and prior computational modeling suggests that heightened I_NaP_ underlies the phenotype of increased susceptibility to depolarization block in inhibitory interneurons (11). I_NaP_ also functions as an amplifier of synaptic currents (70, 71), although we did not observe differences in amplitude of uIPSCs in recordings of synaptically connected pairs. Because I_NaP_ is a consistent, non-inactivating component of the sodium current (44), we hypothesize that elevations in I_NaP_ contribute to premature failure of PV interneurons and subsequent entry into depolarization block. Additionally, I_NaR_ currents are crucial in facilitating repetitive, high-frequency firing, as they affect fast inactivation through an open channel block (40, 72), and Na_v_1.6 is a crucial contributor to I_NaR_ (41). We only observed a significant increase in I_NaR_ in *Scn8a*^W/+^-PV interneurons and not in *Scn8a*^D/+^ PV interneurons, possibly due to mutation-specific effects: this has been observed previously in patient-derived neurons (73). Increases in I_NaR_ likely provide excessive depolarizing current resulting in an increase in firing frequencies, which may be responsible for differences observed between *Scn8a*^D/+^ and *Scn8a*^W/+^-PV interneuron firing, as *Scn8a*^D/+^ PV interneurons enter depolarization block at lower current injections.

It is also important to consider the potential consequences of a GOF Na_v_1.6 mutation on the structural composition of the AIS. Sodium channels are expressed together with potassium channels at the AIS, and both play crucial roles in controlling neuronal excitability (8). Further, previous studies suggest interaction between sodium and potassium channels as a result of genetic mutations (74–76). Potassium channels such as K_v_7.2, which is encoded by *KNCQ2*, interacts with Na_v_1.6, and is an important mediator of M-type potassium current (77, 78), or K_v_3.1, which is important for repetitive, high-frequency firing (79), could be affected by these changes in sodium channel function and may underlie some physiological differences observed in *Scn8a* mutant PV interneurons. Interaction between *Scn8a* and *Kcnq2* has been shown previously in a DEE model: in DEE resulting from loss-of-function mutations in *Kcnq2*, an antisense oligonucleotide (ASO) to reduce the expression of *Scn8a* leads to a marked increase in survival (75).

### Implications for SCN8A DEE.

Patients with *SCN8A* variants are typically treated with sodium channel blockers, and many are refractory to treatment, highlighting the need to further understand the basic mechanisms surrounding the *SCN8A* DEE phenotype. Hyperexcitability of excitatory neurons has often been suggested as the underlying cause behind seizures in *SCN8A* DEE, and, contradictory to our results here, a previous study suggests limited involvement of inhibitory interneurons due to the lack of seizures when the R1872W *SCN8A* variant is expressed in all inhibitory interneurons (30). However, in the previous study, the SUDEP phenotype of mice expressing the R1872W variant globally (*Scn8a*^W/+^; EIIa-Cre) is markedly more severe than that of mice expressing the R1872W variant exclusively in forebrain excitatory neurons (*Scn8a*^W/+^; EMX1-Cre), with median survival of 15 days and 46 days, respectively (30), suggesting the involvement of additional cell types. While we acknowledge the critical contributions of excitatory neuron dysfunction to the *SCN8A* DEE phenotype (10, 30, 62), here we provide compelling support for a major involvement of PV inhibitory interneurons in the onset of spontaneous seizures and seizure-induced death in *SCN8A* DEE.

Gene therapies are in development for both *SCN8A* DEE and Dravet syndrome, and downregulation of *Scn8a* has been shown to reduce seizures in both disorders (80–82). Specifically, an ASO for *Scn8a* was able to significantly delay seizure onset and increase survival in mice that express the R1872W *SCN8A* variant globally (80). This ASO treatment targeted both excitatory and inhibitory neurons. Our previous studies have shown that ASO-mediated rescue of PV interneuron firing reduces seizures and prevents SUDEP in a model of Dravet syndrome (82); a similar phenotype may be observed in *SCN8A* DEE, where rescue of depolarization block prevents seizures and SUDEP. In a similar manner to Dravet syndrome, specific targeting of inhibitory interneurons in *SCN8A* DEE may be a novel therapeutic strategy.

In conclusion, we show here that PV interneurons play an important role in *SCN8A* DEE. Elevations in I_NaP_ likely render PV interneurons more susceptible to AP failure, and subsequent depolarization block leads to a decrease in network inhibition. PV interneurons also exhibit impaired synaptic transmission, and together, we observe that a GOF *SCN8A* variant exclusively expressed in PV interneurons conveys susceptibility to spontaneous seizures and SUDEP. In the field of *SCN8A* DEE, prior research has focused primarily on the impact of GOF *SCN8A* mutations on excitatory neurons (10, 30, 62). These results, along with our previous work proposing that SST interneurons contribute to seizures (11), shift the paradigm of the *SCN8A* DEE field from primarily considering excitatory neuron hyperexcitability as the sole driver of the seizure phenotype and call for future studies to further explore the importance of inhibitory neuron activity in *SCN8A* DEE.

## Methods

### Sex as a biological variable.

Both male and female mice were used in this study. While roughly equal numbers of each sex were used in each experimental group, sex was not considered as a biological variable.

### Mouse husbandry and genotyping.

*Scn8a*^D/+^ and *Scn8a*^W/+^ mice were generated as previously described and maintained through crosses with C57BL/6J mice (The Jackson Laboratory [Jax], 000664) to keep all experimental mice on a C57BL/6J genetic background (29, 30). Both *Scn8a*^D/+^ and *Scn8a*^W/+^ transgenic mice were previously gifted from Miriam Meisler at the University of Michigan, Ann Arbor, Michigan, USA. Cell type–specific expression of R1872W was achieved using males heterozygous for the R1872W allele and C57BL/6J females homozygous for PV-Cre (Jax, 017320) to generate mutant mice (*Scn8a*^W/+^-PV) (30). Homozygous PV-IRES-Cre females were used for breeding to ensure minimal germline recombination due to Cre, as shown previously (83, 84). Because certain transgenic mice entail the insertion of Cre directly into the coding sequence and because of the need for a fluorescent reporter to reliably identify PV interneurons in-slice, for all experiments we used WT controls that contained the same Cre allele but lacked the allele encoding the *Scn8a* variant. Fluorescent labeling of PV interneurons was achieved by first crossing *Scn8a*^D/+^ or *Scn8a*^W/+^ mice with C57BL/6J mice homozygous for a Cre-dependent tdTomato reporter (Jax, 007909) to generate *Scn8a*^D/+^ tdTomato or *Scn8a*^W/+^ tdTomato mice. Then, male *Scn8a*^D/+^ tdTomato or *Scn8a*^W/+^ tdTomato mice were crossed with female mice homozygous for PV-Cre. Experimental groups used at least 3 randomly selected mice to achieve statistical power and roughly equal numbers of male and female mice. All genotyping was conducted through Transnetyx automated genotyping PCR services.

### In vivo seizure monitoring.

Custom EEG headsets (PlasticsOne) were implanted in 5-week-old *Scn8a*^W/+^-PV mice and 6- to 8-week-old *Scn8a*^D/+^ mice using standard surgical techniques as previously described (85). Anesthesia was induced with 5% and maintained with 0.5%–3% isoflurane. Adequacy of anesthesia was assessed by lack of toe-pinch reflex. A midline skin incision was made over the skull and connective tissue was removed. Burr holes were made at the lateral/anterior end of the left and right parietal bones to place EEG leads and at the interparietal bone for ground electrodes. EEG leads were placed bilaterally in the cortex or unilaterally placed in the cortex and superior colliculus using a twist. A headset was attached to the skull with dental acrylic (Jet Acrylic; Lang Dental). Mice received postoperative analgesia with ketoprofen (5 mg/kg, i.p.) and 0.9% saline (0.5 mL i.p.) and were allowed to recover a minimum of 2–5 days before seizure-monitoring experiments.

Mice were then individually housed in custom-fabricated chambers and monitored for the duration of the experiment. The headsets were attached to a custom low-torque swivel cable, allowing mice to move freely in the chamber. EEG signals were amplified at 2,000× original magnification, and bandpass-filtered between 0.3 and 100 Hz, with an analog amplifier (Neurodata model 12, Grass Instruments). Biosignals were digitized with a Powerlab 16/35 and recorded using LabChart 7 software at 1 kS/s. Video acquisition was performed by multiplexing 4 miniature night vision–enabled cameras and then digitizing the video feed with a Dazzle Video Capture Device and recording at 30 fps with LabChart 7 software in tandem with biosignals.

### Immunohistochemistry.

Brain tissue for immunohistochemistry was processed as previously described (11, 86). Mice were anesthetized and transcardially perfused with 10 mL PBS followed by 10 mL 4% paraformaldehyde (PFA). Brains were immersed in 4% PFA overnight at 4°C and stored in PBS. Coronal brain sections, 30 μm, were obtained using a cryostat. Sections were incubated with mouse anti-PV (MilliporeSigma, MAB1572) diluted in 2% goat serum (Jackson ImmunoResearch Laboratories) with 0.1% Triton X-100 (MilliporeSigma) at a concentration of 1:500 in Dulbecco’s PBS. The secondary antibody, goat anti-mouse Alexa Fluor 488 (Invitrogen, A-11029), was diluted 1:1,000 in goat serum (2%) and Triton X-100 (0.1%) in Dulbecco’s PBS. Sections were stained free-floating in primary antibody on a shaker at 4°C overnight and with secondary antibody for 1 hour at room temperature the following day. Tissues were counterstained with NucBlue Fixed Cell ReadyProbes Reagent (DAPI) (Thermo Fisher Scientific, catalog R37606) included in the secondary antibody solution. Tissues were mounted on slides using AquaMount (Polysciences).

### Brain slice preparation.

Preparation of acute brain slices for patch-clamp electrophysiology experiments was modified from standard protocols previously described (10, 11, 30). Mice were anesthetized with isoflurane and decapitated. The brains were rapidly removed and kept in chilled artificial cerebrospinal fluid (ACSF) (0°C) containing (in mM): 125 NaCl, 2.5 KCl, 1.25 NaH_2_PO_4_, 2 CaCl_2_, 1 MgCl_2_, 0.5 l-ascorbic acid, 10 glucose, 25 NaHCO_3_, and 2 Na-pyruvate. For dual-cell patch-clamp experiments, the slicing solution was modified to contain (in mM): 93 *N*-Methyl-d-glucamine, 2.5 KCl, 1.25 NaH_2_PO_4_, 20 HEPES, 5 l-ascorbic acid (sodium salt), 2 thiourea, 3 sodium pyruvate, 0.5 CaCl_2_, 10 MgSO_4_, 25 d-glucose, and 12 *N*-acetyl-l-cysteine, 30 NaHCO_3_, with pH adjusted to 7.2–7.4 using HCl (osmolarity 310 mOsm). Slices were continuously oxygenated with 95% O_2_ and 5% CO_2_ throughout the preparation. Coronal or horizontal brain sections, 300 μm, were prepared using a Leica Microsystems VT1200 vibratome. Slices were collected and placed in ACSF warmed to 37°C for about 30 minutes and then kept at room temperature for up to 6 hours.

### Electrophysiology recordings.

Brain slices were placed in a chamber superfused (~2 mL/min) with continuously oxygenated recording solution warmed to 32 ± 1°C. In *Scn8a*^D/+^ tdTomato PV-Cre, *Scn8a*^W/+^ tdTomato PV-Cre, or WT tdTomato PV-Cre mice, cortical layer IV/V PV interneurons were identified as red fluorescent cells, and pyramidal neurons were identified based on morphology and absence of fluorescence via video microscopy using a Carl Zeiss Axioscope microscope. Whole-cell recordings were performed using a Multiclamp 700B amplifier with signals digitized by a Digidata 1322A digitizer. Currents were amplified, lowpass-filtered at 2 kHz, and sampled at 100 kHz. Borosilicate electrodes were fabricated using a Brown-Flaming puller (model P1000, Sutter Instruments) to have pipette resistances between 1.5 and 3.5 MΩ. All patch-clamp electrophysiology data were analyzed using custom MATLAB scripts or ClampFit 10.7.

### Intrinsic excitability recordings.

Current-clamp recordings of neuronal excitability were collected in ACSF solution identical to that used for preparation of brain slices. The internal solution contained the following (in mM): 120 K-gluconate, 10 NaCl, 2 MgCl_2_, 0.5 K_2_EGTA, 10 HEPES, 4 Na_2_ATP, and 0.3 NaGTP, pH 7.2 (osmolarity 290 mOsm). Intrinsic excitability was assessed using methods adapted from those previously described (10, 11). Briefly, resting membrane potential was manually recorded from the neuron at rest. Current ramps from 0 to 400 pA over 4 seconds were used to calculate passive membrane and AP properties, including threshold, upstroke and downstroke velocity, which are the maximum and minimum slopes on the AP, respectively; amplitude, which was defined as the voltage range between AP peak and threshold; APD_50_, which is the duration of the AP at the midpoint between threshold and peak; input resistance, which was calculated using a –20 pA pulse in current-clamp recordings; and rheobase, which was defined as the maximum amount of depolarizing current that could be injected into neurons before eliciting an AP. AP frequency–current relationships were determined using 1-second current injections from –140 to 1,200 pA. Spikes were only counted if AP overshoot was >0 mV and amplitude was >20 mV. The threshold for depolarization block was operationally defined as the current injection step that elicited the maximum number of APs (i.e., subsequent current injection steps of greater magnitude resulted in fewer APs because of entry into depolarization block).

### Sodium current recordings.

I_NaP_ and I_NaR_ were recorded in the whole-cell patch clamp configuration in-slice, whereas transient sodium current was recorded in the outside-out configuration. The internal solution for all voltage-gated sodium channel recordings contained the following (in mM): 140 CsF, 2 MgCl_2_, 1 EGTA, 10 HEPES, 4 Na_2_ATP, and 0.3 NaGTP with the pH adjusted to 7.3 and osmolality to 300 mOsm. The external solution for recording persistent and resurgent sodium currents has been previously described (87, 88) and contained (in mM): 100 NaCl, 40 TEACl, 10 HEPES, 3.5 KCl, 2 CaCl_2_, 2 MgCl_2_, 0.2 CdCl_2_, 4 of 4-aminopyridine, and 25 d-glucose. Outside-out recordings of transient sodium current were collected in ACSF as the external solution. Steady-state I_NaP_s were elicited using a voltage ramp (20 mV/s) from –80 to –20 mV. To record resurgent sodium currents (I_NaR_), PV interneurons were held at –100 mV, depolarized to 30 mV for 20 ms, and then stepped to voltages between −100 mV and 0 mV for 40 ms. After collecting recordings at baseline, protocols were repeated in the presence of 500 nM TTX (Alomone Labs) to completely block I_NaP_ and I_NaR_. Traces obtained in the presence of TTX were subtracted from those obtained in its absence. The V_1/2_ of I_NaP_ was calculated as previously described (88). Patch clamp recordings in the outside-out configuration were collected using a protocol modified from an approach previously described (10, 11). Voltage-dependent activation and steady-state inactivation parameters were recorded using voltage protocols previously described (11). For all sodium current recordings, we waited 2 minutes after achieving whole-cell configuration to account for initial shifts in the voltage dependence of activation.

### IPSC recordings.

Patch-clamp recordings of IPSCs generated in PCs were performed using the same ACSF external solution and an internal solution containing (in mM): 70 K-Gluconate, 70 KCl, 10 HEPES, 1 EGTA, 2 MgCl_2_, 4 MgATP, and 0.3 Na_3_GTP, with the pH adjusted to 7.2–7.4 and osmolarity to 290 mOsm. Pyramidal cells were held at –70 mV and a 1-minute gap-free recording was performed in the voltage-clamp configuration to assess spontaneous IPSC frequencies before bath application of 500 nM TTX to record miniature IPSCs. After recording spontaneous and miniature IPSCs, 1 μM gabazine was bath applied to block currents and ensure that only inhibitory events were recorded.

### Dual-cell synaptic connection recordings.

uIPSCs were obtained via 2 simultaneous patch-clamp recordings from synaptically connected neurons located within 50 μm of one another in the somatosensory cortex of a horizontal slice. A 2 ms pulse at 1,000 pA elicited APs in the presynaptic neuron at 1, 5, 10, 20, 40, 80, and 120 Hz. The internal solution was modified to contain (in mM): 65 K-gluconate, 65 KCl, 2 MgCl_2_, 10 HEPES, 0.5 EGTA, 10 phosphocreatine-Tris_2_, 4 MgATP, and 0.3 NaGTP, with pH adjusted to 7.2–7.4 using KOH (osmolarity 290 mOsm) (23). PPR was calculated as the amplitude of the second IPSC divided by the amplitude of the first IPSC. PPR was not calculated for trials in which the first and/or second IPSC event was a failure. uIPSC failures were identified by the absence of a transient current greater than 5 pA occurring within 5 ms of the presynaptic AP. Synaptically connected pairs were not used for analysis if resting membrane potential shifted more than 10 mV during recording.

### Statistics.

Analysis of electrophysiological data was performed in a blinded manner. All statistical comparisons were made using the appropriate test in GraphPad Prism 9. Categorical data were analyzed using Fisher’s exact test. For membrane and AP properties, spontaneous firing frequency, depolarization block threshold, peak sodium currents, half-maximal voltages, IPSC frequency and amplitude, and synaptic uIPSC properties, mouse genotypes were compared by 1-way ANOVA followed by Dunnett’s multiple comparisons test when the data were normally distributed with equal variances, by Brown-Forsythe ANOVA with Dunnett’s multiple comparisons test when the data were normally distributed with unequal variances, and by the nonparametric Kruskal–Wallis test followed by Dunn’s multiple comparisons test when the data were not normally distributed. Data were assessed for normality using the Shapiro-Wilk test. Bartlett’s test with *P* = 0.05 was used to assess equal variance. Data were tested for outliers using the ROUT or Grubbs’s method to identify outliers, and statistical outliers were not included in data analysis. A 2-way ANOVA followed by Tukey’s test for multiple comparisons was used to compare groups in experiments in which repetitive measures were made from a single cell over various voltage commands or current injections. Cumulative distribution (survival) plots were analyzed by the log-rank Mantel-Cox test. Data are presented as individual data points and/or mean ± SEM. Exact *n* and *P* values are reported in figure legends.

### Study approval.

Animal experiments were performed in compliance with animal care guidelines issued by the NIH and Animal Care and Use Committee at the University of Virginia (protocol approval no. 3308).

### Data availability.

Individual data values are available in the Supporting Data Values document. Supplemental material is available in the online version.

## Author contributions

RMM, ERW, and MKP conceptualized the study. RMM, ARB, SK, JCH, PSP, MSY, TCJD, CMR, SRV, and ERW acquired data for the study. RMM, ARB, SK, and MSY analyzed data for the study. RMM compiled all figures for the manuscript. RMM and MKP drafted the manuscript. RMM, ERW, and MKP edited the manuscript.

## Supplementary Material

Supplemental data

Supplemental video 1

Supplemental video 2

Supplemental video 3

Supporting data values

## Figures and Tables

**Table 1:**
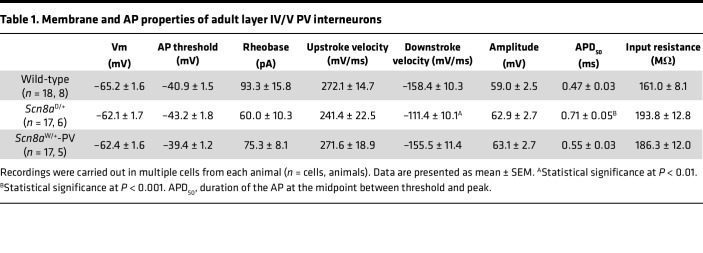
Membrane and AP properties of adult layer IV/V PV interneurons

**Table 2:**
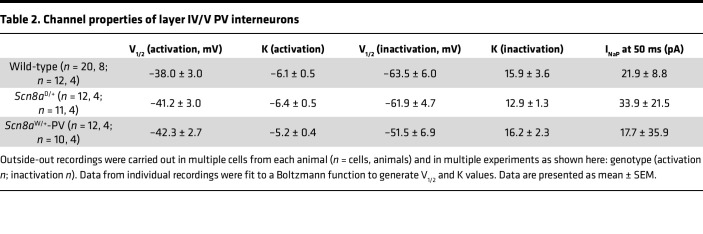
Channel properties of layer IV/V PV interneurons

**Table 3:**
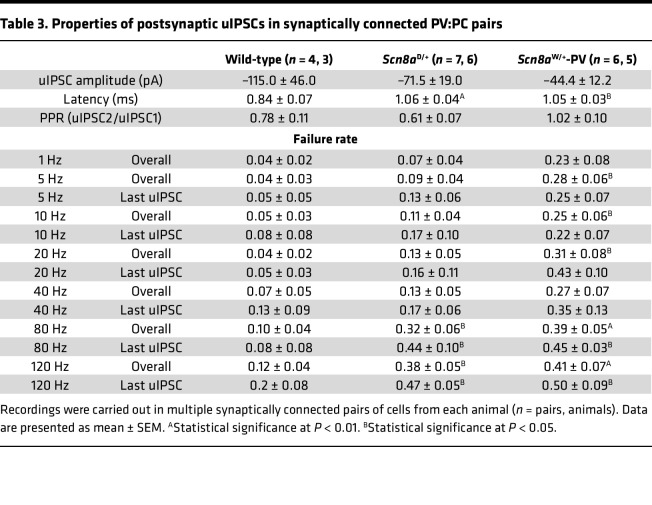
Properties of postsynaptic uIPSCs in synaptically connected PV:PC pairs

## Version 1 10/22/2024

Electronic publication

## References

[1] Meisler M (2019). SCN8A encephalopathy: mechanisms and models. Epilepsia.

[2] Talwar D, Hammer MF (2021). SCN8A epilepsy, developmental encephalopathy, and related disorders. Pediatr Neurol.

[3] Larsen J (2015). The phenotypic spectrum of SCN8A encephalopathy. Neurology.

[4] Gardella E, Møller RS (2019). Phenotypic and genetic spectrum of SCN8A-related disorders, treatment options, and outcomes. Epilepsia.

[5] Veeramah KR (2012). De novo pathogenic SCN8A mutation identified by whole-genome sequencing of a family quartet affected by infantile epileptic encephalopathy and SUDEP. Am J Hum Genet.

[6] Burgess DL (1995). Mutation of a new sodium channel gene, Scn8a, in the mouse mutant ‘motor endplate disease’. Nat Genet.

[7] Caldwell JH (2000). Sodium channel Nav1.6 is localized at nodes of Ranvier, dendrites, and synapses. Proc Natl Acad Sci U S A.

[8] Lorincz A, Nusser Z (2008). Cell-type-dependent molecular composition of the axon initial segment. J Neurosci.

[9] Li T (2014). Action potential initiation in neocortical inhibitory interneurons. PLoS Biol.

[10] Ottolini M (2017). Aberrant sodium channel currents and hyperexcitability of medial entorhinal cortex neurons in a mouse model of *SCN8A* encephalopathy. J Neurosci.

[11] Wengert ER (2021). Somatostatin-positive interneurons contribute to seizures in *SCN8A* epileptic encephalopathy. J Neurosci.

[12] Bernard C (2000). What is GABAergic inhibition? How is it modified in epilepsy?. Epilepsia.

[13] Kumar SS, Buckmaster PS (2006). Hyperexcitability, interneurons, and loss of GABAergic synapses in entorhinal cortex in a model of temporal lobe epilepsy. J Neurosci.

[14] Tremblay R (2016). GABAergic interneurons in the neocortex: from cellular properties to circuits. Neuron.

[15] Rudy B (2011). Three groups of interneurons account for nearly 100% of neocortical GABAergic neurons. Dev Neurobiol.

[16] Gouwens NW (2020). Integrated morphoelectric and transcriptomic classification of cortical GABAergic cells. Cell.

[17] Tasic B (2018). Shared and distinct transcriptomic cell types across neocortical areas. Nature.

[18] Bhattacherjee A (2023). Spatial transcriptomics reveals the distinct organization of mouse prefrontal cortex and neuronal subtypes regulating chronic pain. Nat Neurosci.

[19] Paul A (2017). Transcriptional architecture of synaptic communication delineates GABAergic neuron identity. Cell.

[20] Tai C (2014). Impaired excitability of somatostatin- and parvalbumin-expressing cortical interneurons in a mouse model of Dravet syndrome. Proc Natl Acad Sci U S A.

[21] Favero M (2018). A transient developmental window of fast-spiking interneuron dysfunction in a mouse model of dravet syndrome. J Neurosci.

[22] Tran CH (2020). Interneuron desynchronization precedes seizures in a mouse model of dravet syndrome. J Neurosci.

[23] Kaneko K (2022). Developmentally regulated impairment of parvalbumin interneuron synaptic transmission in an experimental model of Dravet syndrome. Cell Rep.

[24] Bouilleret V (2000). Early loss of interneurons and delayed subunit-specific changes in GABA(A)-receptor expression in a mouse model of mesial temporal lobe epilepsy. Hippocampus.

[25] van Vliet EA (2004). Progression of temporal lobe epilepsy in the rat is associated with immunocytochemical changes in inhibitory interneurons in specific regions of the hippocampal formation. Exp Neurol.

[26] Bausch SB (2005). Axonal sprouting of GABAergic interneurons in temporal lobe epilepsy. Epilepsy Behav.

[27] Wittner L, Maglóczky Z (2017). Synaptic reorganization of the perisomatic inhibitory network in hippocampi of temporal lobe epileptic patients. Biomed Res Int.

[28] Jones JM, Meisler MH (2014). Modeling human epilepsy by TALEN targeting of mouse sodium channel Scn8a. Genesis.

[29] Wagnon JL (2015). Convulsive seizures and SUDEP in a mouse model of SCN8A epileptic encephalopathy. Hum Mol Genet.

[30] Bunton-Stasyshyn RKA (2019). Prominent role of forebrain excitatory neurons in SCN8A encephalopathy. Brain.

[31] Wenker IC (2021). Postictal death is associated with tonic phase apnea in a mouse model of sudden unexpected death in epilepsy. Ann Neurol.

[32] Berecki G (2019). SCN1A gain of function in early infantile encephalopathy. Ann Neurol.

[33] Ziburkus J (2006). Interneuron and pyramidal cell interplay during in vitro seizure-like events. J Neurophysiol.

[34] Cammarota M (2013). Fast spiking interneuron control of seizure propagation in a cortical slice model of focal epilepsy. J Physiol.

[35] Kim CM, Nykamp DQ (2017). The influence of depolarization block on seizure-like activity in networks of excitatory and inhibitory neurons. J Comput Neurosci.

[36] Călin A (2021). Disrupting epileptiform activity by preventing parvalbumin interneuron depolarization block. J Neurosci.

[37] Jayant K (2019). Flexible nanopipettes for minimally invasive intracellular electrophysiology in vivo. Cell Rep.

[38] Rhodes TH (2004). Noninactivating voltage-gated sodium channels in severe myoclonic epilepsy of infancy. Proc Natl Acad Sci U S A.

[39] Raman IM, Bean BP (1997). Resurgent sodium current and action potential formation in dissociated cerebellar Purkinje neurons. J Neurosci.

[40] Khaliq ZM (2003). The contribution of resurgent sodium current to high-frequency firing in Purkinje neurons: an experimental and modeling study. J Neurosci.

[41] Raman IM (1997). Altered subthreshold sodium currents and disrupted firing patterns in Purkinje neurons of Scn8a mutant mice. Neuron.

[42] Hargus NJ (2013). Evidence for a role of Nav1.6 in facilitating increases in neuronal hyperexcitability during epileptogenesis. J Neurophysiol.

[43] Jarecki BW (2010). Human voltage-gated sodium channel mutations that cause inherited neuronal and muscle channelopathies increase resurgent sodium currents. J Clin Invest.

[44] Wengert ER, Patel MK (2020). The role of the persistent sodium current in epilepsy. Epilepsy Curr.

[45] Estacion M (2014). A novel de novo mutation of SCN8A (Nav1.6) with enhanced channel activation in a child with epileptic encephalopathy. Neurobiol Dis.

[46] de Kovel CGF (2014). Characterization of a de novo SCN8A mutation in a patient with epileptic encephalopathy. Epilepsy Res.

[47] Wagnon JL (2016). Pathogenic mechanism of recurrent mutations of SCN8A in epileptic encephalopathy. Ann Clin Transl Neurol.

[48] Fatt P, Katz B (1952). Spontaneous subthreshold activity at motor nerve endings. J Physiol.

[49] Ropert N (1990). Characteristics of miniature inhibitory postsynaptic currents in CA1 pyramidal neurones of rat hippocampus. J Physiol.

[50] Ma Y, Prince DA (2012). Functional alterations in GABAergic fast-spiking interneurons in chronically injured epileptogenic neocortex. Neurobiol Dis.

[51] Rossignol E (2013). CaV 2.1 ablation in cortical interneurons selectively impairs fast-spiking basket cells and causes generalized seizures. Ann Neurol.

[52] Hu H, Jonas P (2014). A supercritical density of Na(+) channels ensures fast signaling in GABAergic interneuron axons. Nat Neurosci.

[53] Kraushaar U, Jonas P (2000). Efficacy and stability of quantal GABA release at a hippocampal interneuron–principal neuron synapse. J Neurosci.

[54] Caillard O (2000). Role of the calcium-binding protein parvalbumin in short-term synaptic plasticity. Proc Natl Acad Sci U S A.

[55] Xiang Z (2002). Synaptic inhibition of pyramidal cells evoked by different interneuronal subtypes in layer v of rat visual cortex. J Neurophysiol.

[56] Katz B, Miledi R (1965). The measurement of synaptic delay, and the time course of acetylcholine release at the neuromuscular junction. Proc R Soc Lond B Biol Sci.

[57] Sabatini BL, Regehr WG (1999). Timing of synaptic transmission. Annu Rev Physiol.

[58] Boudkkazi S (2007). Release-dependent variations in synaptic latency: a putative code for short- and long-term synaptic dynamics. Neuron.

[59] Boudkkazi S (2011). Presynaptic action potential waveform determines cortical synaptic latency. J Physiol.

[60] Pouille F, Scanziani M (2001). Enforcement of temporal fidelity in pyramidal cells by somatic feed-forward inhibition. Science.

[61] Rubinstein M (2015). Dissecting the phenotypes of Dravet syndrome by gene deletion. Brain.

[62] Lopez-Santiago LF (2017). Neuronal hyperexcitability in a mouse model of *SCN8A* epileptic encephalopathy. Proc Natl Acad Sci U S A.

[63] Yuan Y (2024). Antisense oligonucleotides restore excitability, GABA signalling and sodium current density in a Dravet syndrome model. Brain.

[64] Bartos M (2007). Synaptic mechanisms of synchronized gamma oscillations in inhibitory interneuron networks. Nat Rev Neurosci.

[65] Lapray D (2012). Behavior-dependent specialization of identified hippocampal interneurons. Nat Neurosci.

[66] Buzsáki G, Draguhn A (2004). Neuronal oscillations in cortical networks. Science.

[67] Baker SN (2007). Oscillatory interactions between sensorimotor cortex and the periphery. Curr Opin Neurobiol.

[68] Cheah CS (2021). Sharp-wave ripple frequency and interictal epileptic discharges increase in tandem during thermal induction of seizures in a mouse model of genetic epilepsy. Front Cell Neurosci.

[69] Hargus NJ (2011). Temporal lobe epilepsy induces intrinsic alterations in Na channel gating in layer II medial entorhinal cortex neurons. Neurobiol Dis.

[70] Schwindt PC, Crill WE (1995). Amplification of synaptic current by persistent sodium conductance in apical dendrite of neocortical neurons. J Neurophysiol.

[71] Stuart G (1999). Voltage–activated sodium channels amplify inhibition in neocortical pyramidal neurons. Nat Neurosci.

[72] Raman IM, Bean BP (2001). Inactivation and recovery of sodium currents in cerebellar purkinje neurons: evidence for two mechanisms. Biophys J.

[73] Tidball AM (2020). Variant-specific changes in persistent or resurgent sodium current in SCN8A-related epilepsy patient-derived neurons. Brain.

[74] Spratt PWE (2021). Paradoxical hyperexcitability from Na_V_1.2 sodium channel loss in neocortical pyramidal cells. Cell Rep.

[75] Hill SF (2022). Genetic interaction between Scn8a and potassium channel genes Kcna1 and Kcnq2. Epilepsia.

[76] Zhang J (2021). Severe deficiency of the voltage-gated sodium channel Na_V_1.2 elevates neuronal excitability in adult mice. Cell Rep.

[77] Pablo JL, Pitt GS (2017). FGF14 is a regulator of KCNQ2/3 channels. Proc Natl Acad Sci U S A.

[78] Jentsch TJ (2000). Neuronal KCNQ potassium channels: physiology and role in disease. Nat Rev Neurosci.

[79] Rudy B, McBain CJ (2001). Kv3 channels: voltage-gated K+ channels designed for high-frequency repetitive firing. Trends Neurosci.

[80] Lenk GM (2020). Scn8a antisense oligonucleotide is protective in mouse models of SCN8A encephalopathy and dravet syndrome. Ann Neurol.

[81] Han Z (2020). Antisense oligonucleotides increase *Scn1a* expression and reduce seizures and SUDEP incidence in a mouse model of Dravet syndrome. Sci Transl Med.

[82] Wengert ER (2022). Targeted Augmentation of Nuclear Gene Output (TANGO) of Scn1a rescues parvalbumin interneuron excitability and reduces seizures in a mouse model of Dravet Syndrome. Brain Res.

[83] Kobayashi Y, Hensch T (2013). Germline recombination by conditional gene targeting with Parvalbumin-Cre lines. Front Neural Circuits.

[84] Luo L (2020). Optimizing nervous system-specific gene targeting with Cre driver lines: prevalence of germline recombination and influencing factors. Neuron.

[85] Wenker IC (2022). Forebrain epileptiform activity is not required for seizure-induced apnea in a mouse model of Scn8a epilepsy. Front Neural Circuits.

[86] Thompson JA (2022). Astrocyte reactivity in a mouse model of SCN8A epileptic encephalopathy. Epilepsia Open.

[87] Royeck M (2008). Role of axonal NaV1.6 sodium channels in action potential initiation of CA1 pyramidal neurons. J Neurophysiol.

[88] Wengert ER (2019). Prax330 reduces persistent and resurgent sodium channel currents and neuronal hyperexcitability of subiculum neurons in a mouse model of SCN8A epileptic encephalopathy. Neuropharmacology.

