# Supplementary material for: Parvalbumin interneuron impairment causes synaptic transmission deficits and seizures in SCN8A developmental and epileptic encephalopathy
Source: JCI Insight. 2024 Oct 22;9(20):e181005. doi: 10.1172/jci.insight.181005 (PMC11529981; doi:10.1172/jci.insight.181005)
Supplement: Supplemental data [file jciinsight-9-181005-s110.pdf]

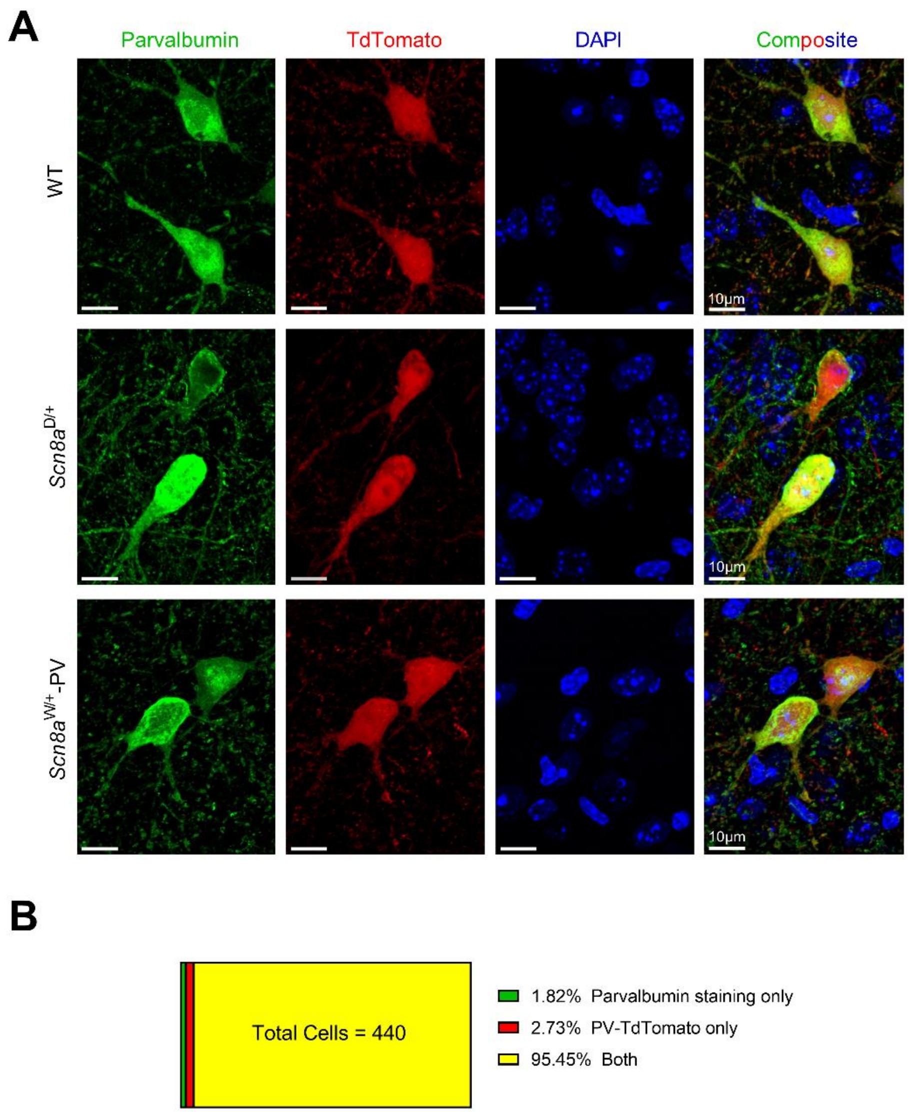

Supplementary Figure 1: Colocalization of parvalbumin and TdTomato in WT, *Scn8a*$^{D/+}$, and *Scn8a*$^{W/+}$-PV mice. (**A**)) Example immunohistochemistry images showing colocalization of parvalbumin (green) and TdTomato (red) immunofluorescence in adult WT, *Scn8a*$^{D/+}$, and *Scn8a*$^{W/+}$-PV mice. Scale bar 10 µM. Images were collected from layer IV/V of the somatosensory cortex in n=3 mice from each genotype. (**B**) Cell counting shows that >95% of cells expressed both parvalbumin and TdTomato.

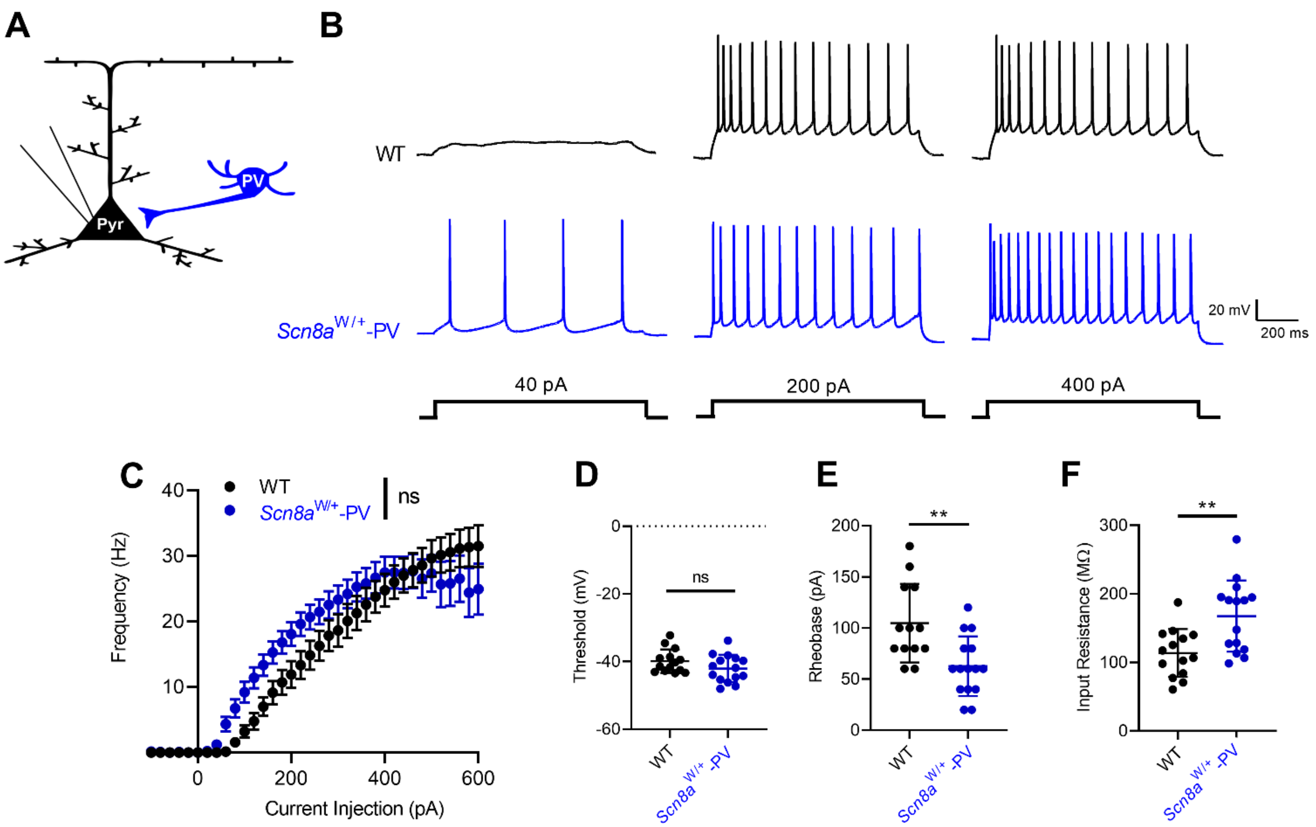

Supplementary Figure 2: Intrinsic excitability of pyramidal cells in *Scn8a*$^{W/+}$-PV mice. (**A**) Whole-cell recordings were collected from pyramidal cells in layer IV/V somatosensory cortex in adult, 5-8 week old WT and *Scn8a*$^{W/+}$-PV mice. (**B**) Example traces of WT (black) and *Scn8a*$^{W/+}$-PV(blue) pyramidal cell firing at 40, 200, and 400 pA current injections. (**C**) Pyramidal cell firing does not significantly differ between WT (*n*=14, 5 mice) and *Scn8a*$^{W/+}$-PV mice (*n*=15, 3 mice, *p*>0.05, 2-way ANOVA). (**D**) There is no significant difference in pyramidal cell AP threshold in WT and *Scn8a*$^{W/+}$-PV mice (*p*<0.05, unpaired t-test). (**E**) Rheobase in *Scn8a*$^{W/+}$-PV pyramidal cells is significantly lower than WT (**, *p*<0.01, unpaired t-test). (**F**) Pyramidal cell input resistance is significantly increased in *Scn8a*$^{W/+}$-PV mice (**, *p*<0.01, unpaired t-test).

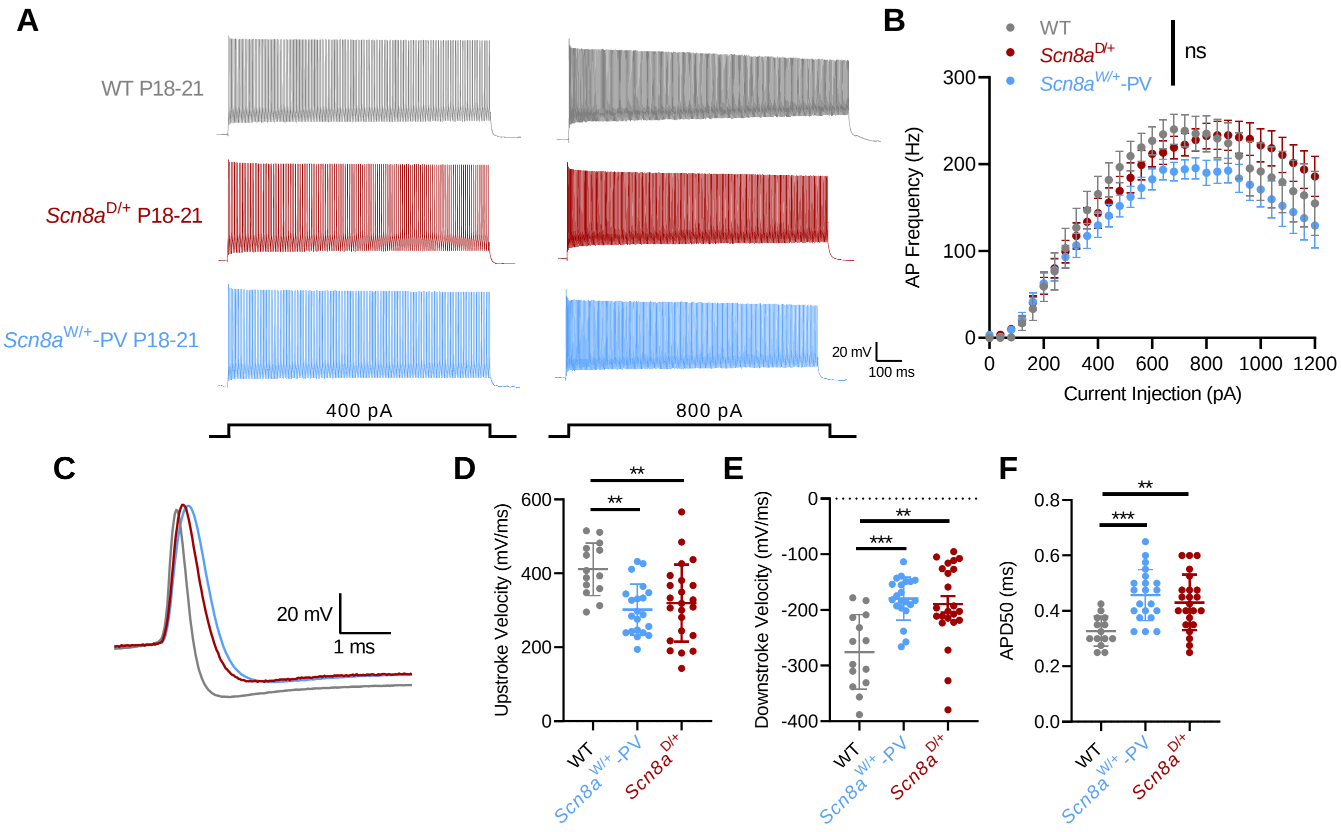

Supplementary Figure 3: Intrinsic excitability of PV interneurons in P18-21 WT, *Scn8a*$^{D/+}$, and *Scn8a*$^{W/+}$-PV mice. (**A**) Example traces of P18-21 PV interneuron firing in WT (gray), *Scn8a*$^{D/+}$ (red), and *Scn8a*$^{W/+}$-PV (blue) mice at 400 and 800 pA current injections. (**B**) PV interneuron firing does not significantly differ between WT (*n*=14, 3 mice), *Scn8a*$^{D/+}$ (*n*=21, 4 mice), and *Scn8a*$^{W/+}$-PV mice (*n*=23, 4 mice, *p*>0.05, 2-way ANOVA) at P18-21. (**C**) Example of a single AP from WT, *Scn8a*$^{D/+}$, and *Scn8a*$^{W/+}$-PV interneuron. (**D**) Upstroke velocity is significantly decreased in *Scn8a*$^{D/+}$ (**, *p*<0.01) and *Scn8a*$^{W/+}$-PV (**, *p*<0.01) interneurons compared to WT (one-way ANOVA with Tukey's multiple comparison test). (**E**) Downstroke velocity is significantly decreased in *Scn8a*$^{D/+}$ (**, *p*<0.01) and *Scn8a*$^{W/+}$-PV (***, *p*<0.001) interneurons compared to WT (Kruskal-Wallis test with Dunn's multiple comparison test). (**F**) *Scn8a*$^{D/+}$ (**, *p*<0.01) and *Scn8a*$^{W/+}$-PV (***, *p*<0.001) interneurons have wider APs than their WT counterparts (one-way ANOVA with Tukey's multiple comparison test).

Supplementary Table 1: Membrane and Action Potential Properties of Adult Layer IV/V Pyramidal Neurons

	**Vm (mV)**	**AP threshold (mV)**	**Rheobase (pA)**	**Upstroke Velocity (mV/ms)**	**Downstroke Velocity (mV/ms)**	**Amplitude (mV)**	**APD_{50} (ms)**	**Input Resistance (MΩ)**
Wild-type (*n*=14, 5)	-64.4 ± 1.3	-39.9 ± 0.9	104.6 ± 10.7	274.1 ± 18.1	-71.0 ± 8.4	84.2 ± 2.5	1.36 ± 0.14	113.8 ± 9.3
Scn8a$^{W/+}$-PV (*n*=15, 3)	-67.6 ± 1.4	-42.1 ± 1.0	*62.7 ± 7.5* **	267.3 ± 21.6	-63.7 ± 6.5	85.6 ± 3.0	1.49 ± 0.16	*167.7 ± 13.4* **

Recordings were carried out in multiple cells from each animal (*n*=cells, animals). Data are presented as mean ± SEM.

* Statistical significance at $P < 0.05$

** Statistical significance at $P < 0.01$

Supplementary Table 2: Membrane and Action Potential Properties of P18-21 Layer IV/V PV Interneurons

	Vm (mV)	AP threshold (mV)	Rheobase (pA)	Upstroke Velocity (mV/ms)	Downstroke Velocity (mV/ms)	Amplitude (mV)	APD_{50} (ms)	Input Resistance (MΩ)
Wild-type (*n*=14, 3)	-66.4 ± 1.1	-37.3 ± 1.1	208.6 ± 20.4	411.5 ± 19.0	-275.4 ± 17.8	66.3 ± 2.0	0.33 ± 0.01	104.0 ± 7.8
Scn8a$^{D/+}$ (*n*=21, 4)	-65.2 ± 1.3	-39.6 ± 1.2	149.5 ± 15.1	*319.6 ± 21.7 ** *	*-189.7 ± 15.0 ** *	61.2 ± 2.6	*0.43 ± 0.02 ** *	106.9 ± 6.2
Scn8a$^{W/+}$-PV (*n*=23, 3)	-64.8 ± 0.8	-36.9 ± 0.9	203.8 ± 24.0	*302.0 ± 15.0 ** *	*-179.5 ± 8.4 *** *	62.1 ± 2.6	*0.46 ± 0.02 *** *	116.2 ± 7.9

Recordings were carried out in multiple cells from each animal (*n*=cells, animals). Data are presented as mean ± SEM.

* Statistical significance at $P < 0.05$

** Statistical significance at $P < 0.01$

*** Statistical significance at $P < 0.001$
